# Identifying adults at high-risk for change in weight and BMI in England: a longitudinal, large-scale, population-based cohort study using electronic health records

**DOI:** 10.1016/S2213-8587(21)00207-2

**Published:** 2021-10

**Authors:** Michail Katsoulis, Alvina G Lai, Karla Diaz-Ordaz, Manuel Gomes, Laura Pasea, Amitava Banerjee, Spiros Denaxas, Kostas Tsilidis, Pagona Lagiou, Gesthimani Misirli, Krishnan Bhaskaran, Goya Wannamethee, Richard Dobson, Rachel L Batterham, Dimitra-Kleio Kipourou, R Thomas Lumbers, Lan Wen, Nick Wareham, Claudia Langenberg, Harry Hemingway

**Affiliations:** a Institute of Health Informatics, University College London, London, UK; b Health Data Research UK, University College London, London, UK; c Department of Applied Health Research, University College London, London, UK; d Department of Primary Care and Population Health, University College London, London, UK; e Institute of Health Informatics, University College London, London, UK; f Centre for Obesity Research, University College London, London, UK; g Department of Medical Statistics, London School of Hygiene and Tropical Medicine, London, UK; h Department of Non-Communicable Disease Epidemiology, London School of Hygiene and Tropical Medicine, London, UK; i University College London Hospitals NHS Trust, London, UK; j Barts Health NHS Trust, The Royal London Hospital, London, UK; k Alan Turing Institute, London, UK; l National Institute of Health Research, University College London Hospitals Biomedical Research Centre, London, UK; m Department of Epidemiology and Biostatistics, School of Public Health, Imperial College London, London, UK; n Department of Hygiene and Epidemiology, University of Ioannina School of Medicine, Ioannina, Greece; o Department of Hygiene, Epidemiology and Medical Statistics, School of Medicine, National and Kapodistrian University of Athens, Athens, Greece; p Department of Epidemiology, Harvard TH Chan School of Public Health, Boston, MA, USA; q Hellenic Health Foundation, Athens, Greece; r Department of Biostatistics and Health Informatics, Institute of Psychiatry, Psychology and Neuroscience, King's College London, London, UK; s University College London Hospitals Bariatric Centre for Weight Management and Metabolic Surgery, London, UK; t MRC Epidemiology Unit, University of Cambridge School of Clinical Medicine, Cambridge, UK; u Computational Medicine, Berlin Institute of Health, Charité–University Medicine Berlin, Berlin, Germany

## Abstract

**Background:**

Targeted obesity prevention policies would benefit from the identification of population groups with the highest risk of weight gain. The relative importance of adult age, sex, ethnicity, geographical region, and degree of social deprivation on weight gain is not known. We aimed to identify high-risk groups for changes in weight and BMI using electronic health records (EHR).

**Methods:**

In this longitudinal, population-based cohort study we used linked EHR data from 400 primary care practices (via the Clinical Practice Research Datalink) in England, accessed via the CALIBER programme. Eligible participants were aged 18–74 years, were registered at a general practice clinic, and had BMI and weight measurements recorded between Jan 1, 1998, and June 30, 2016, during the period when they had eligible linked data with at least 1 year of follow-up time. We calculated longitudinal changes in BMI over 1, 5, and 10 years, and investigated the absolute risk and odds ratios (ORs) of transitioning between BMI categories (underweight, normal weight, overweight, obesity class 1 and 2, and severe obesity [class 3]), as defined by WHO. The associations of demographic factors with BMI transitions were estimated by use of logistic regression analysis, adjusting for baseline BMI, family history of cardiovascular disease, use of diuretics, and prevalent chronic conditions.

**Findings:**

We included 2 092 260 eligible individuals with more than 9 million BMI measurements in our study. Young adult age was the strongest risk factor for weight gain at 1, 5, and 10 years of follow-up. Compared with the oldest age group (65–74 years), adults in the youngest age group (18–24 years) had the highest OR (4·22 [95% CI 3·86–4·62]) and greatest absolute risk (37% *vs* 24%) of transitioning from normal weight to overweight or obesity at 10 years. Likewise, adults in the youngest age group with overweight or obesity at baseline were also at highest risk to transition to a higher BMI category; OR 4·60 (4·06–5·22) and absolute risk (42% *vs* 18%) of transitioning from overweight to class 1 and 2 obesity, and OR 5·87 (5·23–6·59) and absolute risk (22% *vs* 5%) of transitioning from class 1 and 2 obesity to class 3 obesity. Other demographic factors were consistently less strongly associated with these transitions; for example, the OR of transitioning from normal weight to overweight or obesity in people living in the most socially deprived versus least deprived areas was 1·23 (1·18–1·27), for men versus women was 1·12 (1·08–1·16), and for Black individuals versus White individuals was 1·13 (1·04–1·24). We provide an open access online risk calculator, and present high-resolution obesity risk charts over a 1-year, 5-year, and 10-year follow-up period.

**Interpretation:**

A radical shift in policy is required to focus on individuals at the highest risk of weight gain (ie, young adults aged 18–24 years) for individual-level and population-level prevention of obesity and its long-term consequences for health and health care.

**Funding:**

The British Hearth Foundation, Health Data Research UK, the UK Medical Research Council, and the National Institute for Health Research.

## Introduction

Adult obesity prevention policies, which are largely untargeted, have had limited success globally,^1^, ^2^ and the high prevalence of obesity is predicted to increase substantially over the next decade.^3^, ^4^ Population-wide approaches to obesity prevention could be complemented by new targeted approaches if population groups with the highest risk of weight gain could be identified using readily available information in national public health systems.^1^ Current obesity prevention policies are informed by evidence from cross-sectional, population-based surveys,^5^ which, by definition, cannot evaluate weight changes, and highlight the increased cross-sectional prevalence of obesity in middle-aged individuals (ie, those aged 40–59 years), those residing in socially deprived areas, and in particular ethnic groups.^6^ In longitudinal research, younger adults have been shown to have a greater risk of weight gain than older adults in many,^7^, ^8^, ^9^ but not all, studies (appendix pp 3–4).^10^

**Research in context**

**Evidence before this study**

Adult obesity prevention policies have had limited success globally, and have thus far not sought to identify and target groups in the population who have the highest risk of weight gain, including those of a normal weight (ie, a BMI of 18·5–24·9 kg/m^2^), overweight (ie, a BMI of 25·0–29·9 kg/m^2^), and obesity (ie, a BMI of ≥30·0 kg/m^2^). We searched PubMed on Dec 20, 2020, using the search string “(weight[Title])” OR “(body mass index[Title])” AND “(change)”. We searched for longitudinal population-based studies of intra-individual weight and BMI changes according to age and other demographic factors published between Jan 1, 2000, and Dec 20, 2020. We found 18 studies involving population-based cohorts of intra-individual BMI changes in adults in studies with sufficiently large sample size (at least 5000 participants in total, and at least 1000 young adults; appendix pp 2–3). Young adult age was shown in some, but not all studies to be associated with the highest increase in weight and BMI when compared with older ages. Other studies suggested separate roles for the effects of ethnicity and sex on increases in weight or BMI. However, these previous studies were too small or did not contain the necessary information to assess multiple risk factors. We found three policy-relevant gaps in the knowledge. First, how to identify population groups at highest risk of weight gain is not known; we found no study of BMI changes that evaluated the combined contribution of initial BMI and four important sociodemographic factors (age, sex, socioeconomic status, and ethnicity). Second, we found no previous study evaluating policy-relevant measures of absolute risk of transitioning between normal weight, overweight, and obesity BMI categories; instead, previous studies have reported continuous measures. Finally, we found no previous study that used large-scale data from electronic health records (EHRs) in the general population to study patterns of weight change in people of a normal weight, or in those with overweight or obesity. We found publications supporting the validity of using EHRs to study weight changes among people with obesity or after bariatric surgery.

**Added value of this study**

We address these three policy-relevant knowledge gaps. First, we show that young adults (aged 18–24 years) had a markedly greater risk for transitioning to higher BMI categories than did older age groups (ie, those aged 65–74 years). Compared with age, we found smaller additional contributions to the risk of BMI change, including being male, living in socially deprived neighbourhoods, and being from a Black ethnic background. Second, in by far the largest study done to date (involving more than two million individuals), we provide high-resolution estimates of BMI change across 900 strata defined by age (six groups), sex (two groups), degree of social deprivation (five groups), and initial BMI (15 categories). We provide the first estimates of the risks of transitioning between underweight, normal weight, overweight, and obesity BMI categories at 1, 5, and 10 years (risk charts and an online tool). Finally, we show the value of using longitudinal population-based EHRs to identify and monitor specific population groups at risk of weight gain. Health records offer advantages of being large scale, low cost, and updatable, and of providing policy-relevant insights.

**Implications of all the available evidence**

A radical shift in policy is required to focus on individuals at highest risk of weight gain (ie, young adults) for individual and population-level prevention of obesity and its long-term consequences for health and health care. Particular opportunities for intervention might arise as young adults transition away from living at home, or into education, relationships, and work.

An emerging data science opportunity to identify population groups at high risk for weight gain comes from longitudinal population-based electronic health records (EHRs). Understanding how adult age, sex, ethnicity, geographical region, and degree of social deprivation might together influence the risk of weight changes to identify groups at highest risk of weight gain is essential if targeted policies are to be considered. We found no previous studies providing such evidence. Population-based EHRs offer valid measurements of weight and BMI changes,^11^ and they have potential advantages (compared with consented cohort studies) of sufficient scale to evaluate the joint contribution of several factors, and of providing updatable and low-cost measurements.

We used population-based EHRs to address longstanding calls for monitoring basic population weight data^12^ in the context of weight change by presenting intra-individual 1-year, 5-year, and 10-year patterns in BMI changes, according to sociodemographic factors, with the following objectives: (1) to test the extent to which temporal trends in mean BMI changes between 1998 and 2016 are replicated between EHRs and survey methods;^13^ (2) to compare the extent and distribution of BMI changes across age groups and BMI categories; (3) to estimate the associations between age, sex, degree of social deprivation, ethnicity, and geographical region with transitions across BMI categories; and (4) to produce a risk calculator (both online and in chart-form), showing how these risk factors combine to identify groups at a high risk of transitioning to higher BMI categories.

## Methods

### Study design and participants

In this longitudinal, population-based cohort study, we used clinically recorded measures of height, weight, and BMI from primary care practices in England, accessed through the Clinical Practice Research Datalink. This study was done as part of the CALIBER research platform). CALIBER, led by the UCL Institute of Health Informatics (University College London, London, UK), is an open resource for researchers providing validated EHR phenotyping algorithms and tools for the use of national structured health record data sources. Through the CALIBER platform,^13^ we obtained data from individuals aged 18–74 years registered at 400 primary care practices in England, with BMI measurements recorded between Jan 1, 1998, and June 30, 2016 (see appendix pp 5–8).

The study was approved by the UK Medicines and Healthcare products Regulatory Agency Independent Scientific Advisory Committee (18_010R), under Section 251 of the National Health Service (NHS) Social Care Act 2006, for the use of anonymised records in research without individual participant written consent.

### Data collection

**Figure 1:**
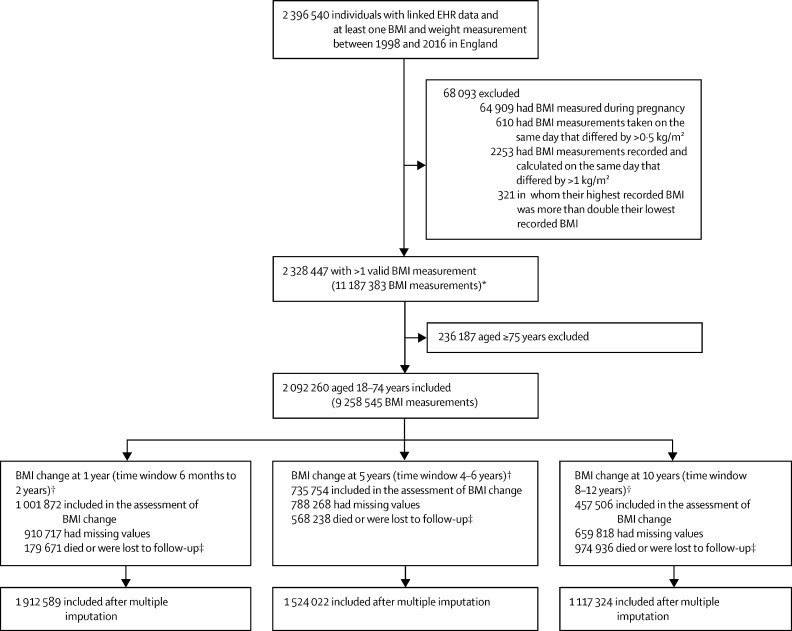
Flow chart showing the number of individuals and BMI measurements recorded in EHRs used for calculating 1-year, 5-year, and 10-year changes in BMI EHR=electronic health record. *Temporal trends in BMI were calculated and compared with HSE data (see appendix pp 6–7). †For people with more than one valid pair of BMI measurements, we chose one pair at random for the calculation of BMI change. ‡We excluded individuals with a follow-up interval since their initial BMI measurement of less than half the time window of interest (ie, <6 months for estimating 1-year BMI changes, 2·5 years for estimating 5-year BMI changes, and 5 years for estimating 10-year BMI changes). §We selected one timepoint with a BMI measurement at random and applied multiple imputation analysis.

NHS policy is for a patient's weight and height to be recorded at registration with a general practice clinic, with these measurements repeated at health checks (currently offered to patients aged 40–74 years and done once every 5 years) and at clinical discretion.^11^ We extracted height (in m), weight (in kg) and BMI (in kg/m^2^) measurements, as recorded during usual care by physicians, nurses, and health-care assistants using a range of devices. We included individuals with BMI and weight measurements recorded during the period in which they had eligible linked data, with at least 1 year of follow-up. We excluded individuals with BMI recorded during pregnancy; who had BMI measurements taken on the same day that differed by more than 0·5 kg/m^2^; whose highest recorded BMI was more than double their lowest recorded BMI; and in whom the absolute difference between recorded and calculated BMI on the same day was more than 1 kg/m^2^ (figure 1). Following WHO guidelines, we defined underweight as a BMI of less than 18·5 kg/m^2^; normal weight as a BMI of 18·5–24·9 kg/m^2^; overweight as a BMI of 25·0–29·9 kg/m^2^; non-severe obesity (class 1 and 2) as a BMI of 30·0–39·9 kg/m^2^; and severe obesity (class 3) as a BMI of 40 kg/m^2^ or higher.

We grouped individuals by age (taken at the date of the first BMI measurement; 18–24 years, 25–34 years, 35–44 years, 45–54 years, 55–64 years, and 65–74 years); sex (male or female); ethnicity (White, Asian [Chinese, Indian, Pakistani, Bangladeshi, or other Asian ethnicity], Black [Black African, Black Caribbean, or other Black ethnicity], or mixed or other ethnicity); degree of social deprivation, measured by use of the region-based Index of Multiple Deprivation (IMD; in quintiles from least deprived [first quintile] to most deprived [fifth quintile]); and geographical region (London, South West England, South Central England, South East England, West Midlands, East of England, central North East England, and North West England). Data on chronic disease, smoking status, and physical activity were also extracted from EHRs.

### Statistical analysis

To compare trends in mean BMI changes over time, we randomly selected one BMI measurement from all eligible individuals included in CALIBER, and compared mean BMI between 1998 and 2016 in England with standardised survey measures of BMI from an annual cross-sectional survey (the Health Survey for England [HSE]), according to age and sex.^14^ The HSE is a source of data, independent of health-care consultation and recording practices, that has been widely used to inform public policy. We also compared EHR measures of BMI with corresponding measures obtained from the US National Health and Nutrition Examination Survey (NHANES)^15^ over the same time period, according to age group.

To estimate 1-year, 5-year, and 10-year changes in BMI, we randomly selected one pair of BMI measurements from individuals who had measurements taken during time windows of 6 months to 2 years, 4 years to 6 years, and 8 years to 12 years, respectively. We assumed that any change in BMI for each patient was linear during each time window (appendix pp 10–12).

The problem of missing values arises because not all individuals had two BMI measurements within the specific time windows of interest (figure 1). We assumed that the missingness mechanism for BMI change was missing not at random. For this reason, we applied multiple imputation with delta-adjustment, as this method provides a flexible and transparent means to impute missing data under missing-not-at-random mechanisms.^16^ We first imputed the missing values, not only for BMI change, but also for other factors using multiple imputation by chained equations (MICE). Specifically, we created ten copies of datasets in which missing values for BMI change, ethnicity, IMD, smoking status, and physical activity were replaced by imputed values sampled from their predictive distribution by use of MICE, which was applied separately for the six age groups. We then added delta values to our imputed datasets of BMI change to minimise any bias due to missingness not at random. After implementation of this approach, the average 10-year BMI change according to age and sex in our study (using CALIBER) and the HSE were the same. More details of the methods used to account for missing data are in the appendix (pp 13–19).

For sociodemographic factors, we estimated the odds ratios (ORs) for transitioning between BMI categories over 1-year, 5-year, and 10-year periods using of logistic regression models. For example, to estimate the ORs for transitioning from overweight to obesity BMI categories, logistic regression was used whereby the dependent variable was “0” for individuals who did not transition and “1” for those who did transition (appendix pp 20–23). In all of these models, we additionally adjusted for BMI at baseline, a family history of cardiovascular disease, use of diuretics, prevalent cardiovascular disease, cancer, diabetes, mental health disorders (depression, anxiety, stress, phobia, schizophrenia, bipolar disorder, or affective disorder), and other prevalent chronic conditions (HIV, chronic obstructive pulmonary disease, neurological disease [dementia], rheumatological disease [rheumatoid arthritis, gout, or systemic lupus erythematosus], gastrointestinal disease [inflammatory bowel disease], or renal disease [chronic kidney disease or renal failure]) at baseline.

We calculated the age-standardised transition between BMI categories at 1, 5, and 10 years on the basis of the age structure of the English population (Office for National Statistics) and BMI information from the HSE (appendix p 24). We produced risk charts estimating the BMI changes at 10 years by age, IMD, and initial BMI, as well as separately for men and women non-parametrically. We also created an online risk calculator (appendix p 25).

### Role of the funding source

The funders of the study had no role in study design, data collection, data analysis, data interpretation, or writing of the report.

## Results

We identified 2 396 540 individuals with linked EHR data and at least one BMI measurement recorded between 1998 and 2016 in England, of whom 2 092 260 individuals aged 18–74 years with at least one valid BMI measurement were included in the analysis (figure 1). The baseline characteristics of all individuals at the time of their first BMI measurements are provided in the table. Regarding the individuals contributing to the assessment of 10-year BMI changes, the proportion of women (646 392 [58%]) was higher than men (470 932 [42%]). Most individuals were White (848 231 [76%]), 99 502 (9%) had prevalent cardiovascular disease, and 44 625 (4%) had prevalent cancer.

**Table:** Baseline characteristics

		**1-year BMI change (n=1 912 589)**	**5-year BMI change (n=1 524 022)**	**10-year BMI change (n=1 117 324)**
Sex				
	Female	1 096 689 (57%)	872 394 (57%)	646 392 (58%)
	Male	815 900 (43%)	651 628 (43%)	470 932 (42%)
Age, years		46·9 (15·5)	47·2 (15·2)	47·4 (14·8)
	18–24	187 574 (10%)	140 510 (9%)	92 779 (8%)
	25–34	289 098 (15%)	210 433 (14%)	146 443 (13%)
	35–44	360 589 (19%)	300 878 (20%)	231 871 (21%)
	45–54	399 105 (21%)	327 372 (21%)	249 941 (22%)
	55–64	365 952 (19%)	305 205 (20%)	231 382 (21%)
	65–74	310 271 (16%)	239 624 (16%)	164 908 (15%)
BMI, kg/m^2^		27·3 (5·9)	27·3 (5·7)	27·3 (5·6)
Distribution of BMI categories at baseline in the 18–24 years age group*				
	Underweight	14 251 (8%)	10 476 (7%)	6740 (7%)
	Normal weight	104 643 (56%)	78 376 (56%)	52 040 (56%)
	Overweight	39 322 (21%)	29 751 (21%)	19 667 (21%)
	Obesity	29 358 (16%)	21 907 (16%)	14 332 (15%)
Distribution of BMI categories at baseline in the 25–34 years age group*				
	Underweight	9481 (3%)	6466 (3%)	4333 (3%)
	Normal weight	135 794 (47%)	97 535 (46%)	67 168 (46%)
	Overweight	80 481 (28%)	59 919 (28%)	42 046 (29%)
	Obesity	63 342 (22%)	46 513 (22%)	32 896 (22%)
Distribution of BMI categories at baseline in the 35–44 years age group*				
	Underweight	5619 (2%)	4402 (1%)	3359 (1%)
	Normal weight	131 898 (37%)	110 404 (37%)	86 130 (37%)
	Overweight	121 598 (34%)	102 260 (34%)	78 455 (34%)
	Obesity	101 474 (28%)	83 812 (28%)	63 927 (28%)
Distribution of BMI categories at baseline in the 45–54-years age group*				
	Underweight	4654 (1%)	3607 (1%)	2636 (1%)
	Normal weight	122 093 (31%)	101 413 (31%)	78 676 (31%)
	Overweight	146 696 (37%)	121 306 (37%)	93 598 (37%)
	Obesity	125 662 (31%)	101 046 (31%)	75 031 (30%)
Distribution of BMI categories at baseline in the 55–64-years age group*				
	Underweight	4603 (1%)	3248 (1%)	2223 (1%)
	Normal weight	103 161 (28%)	87 055 (29%)	67 353 (29%)
	Overweight	142 108 (39%)	121 086 (40%)	93 726 (41%)
	Obesity	116 080 (32%)	93 816 (31%)	68 080 (29%)
Distribution of BMI categories at baseline in the 65–74-years age group*				
	Underweight	5321 (2%)	3208 (1%)	1798 (1%)
	Normal weight	91 674 (30%)	70 856 (30%)	49 000 (30%)
	Overweight	125 115 (40%)	99 644 (42%)	70 734 (43%)
	Obesity	88 161 (28%)	65 916 (28%)	43 376 (26%)
Ethnicity				
	White	1 405 432 (73%)	1 140 068 (75%)	848 231 (76%)
	Black	61 132 (3%)	44 426 (3%)	29 786 (3%)
	Asian	36 260 (2%)	25 418 (2%)	14 893 (1%)
	Mixed or other	23 339 (1%)	16 235 (1%)	10 208 (1%)
	Missing	386 426 (20%)	297 875 (20%)	214 206 (19%)
Index of Multiple Deprivation quintile				
	1 (least deprived)	317 299 (17%)	261 382 (17%)	198 813 (18%)
	2	311 664 (16%)	254 612 (17%)	190 582 (17%)
	3	297 464 (16%)	239 316 (16%)	177 138 (16%)
	4	277 168 (14%)	220 613 (14%)	161 415 (14%)
	5 (most deprived)	244 600 (13%)	192 236 (13%)	137 042 (12%)
	Missing	464 394 (24%)	355 863 (23%)	252 334 (23%)
Family history of cardiovascular disease		484 281 (25%)	403 575 (26%)	312 249 (28%)
Use of diuretics		263 253 (14%)	213 835 (14%)	155 597 (14%)
Prevalence of chronic diseases				
	Cancer	100 838 (5%)	70 018 (5%)	44 625 (4%)
	Cardiovascular disease	184 118 (10%)	141 533 (9%)	99 502 (9%)
	Diabetes	139 737 (7%)	101 046 (7%)	66 601 (6%)
	Hypertension	902 998 (47%)	723 369 (47%)	527 420 (47%)
	Mental health disorders†	580 160 (30%)	433 828 (28%)	298 979 (27%)
	Other chronic diseases‡	200 230 (10%)	140 513 (9%)	86 484 (8%)

Data are n (%) or mean (SD). The table shows baseline characteristics of patients at the time of their first BMI measurement (recorded in their health records), which was used to calculate changes in BMI at 1, 5, and 10 years.

* Percentages are calculated according to the total number of patients in each age group.

† Includes depression, anxiety, stress, phobia, schizophrenia, bipolar disorder, and affective disorder.

‡ Includes chronic obstructive pulmonary disease, neurological disease (dementia), rheumatological disease (rheumatoid arthritis, gout, and systemic lupus erythematosus), gastrointestinal disease (inflammatory bowel disease), HIV, and renal disease (chronic kidney disease and renal failure).

We first validated time trends in mean BMI changes using EHR (CALIBER) data compared with HSE data. Minor differences between mean BMI estimates obtained from EHR and HSE were observed, particularly in individuals aged younger than 65 years (appendix pp 6–7). Mean BMI was higher in older age groups than in younger age groups. We then compared time trends in mean BMI changes using CALIBER with those from US NHANES data. Mean BMI increased in each age group between 1999 and 2016 in England, and the rate of increase was similar to that observed in the USA, with mean BMIs in the USA approximately 2 kg/m^2^ higher in each biannual period and age group compared with England (appendix p 8).

Using EHR data, we estimated the initial and estimated average weight at 10 years according to age and sex (appendix p 28). We found that younger individuals gained more weight on average than older individuals during this time period. For example, the average initial weight in men aged 18–24 years was 80·8 kg, and their average estimated weight after 10 years was 90·2 kg, whereas the average initial weight of men aged 65–74 years was 83·9 kg, and their average estimated weight after 10 years was 82·2 kg.

**Figure 2:**
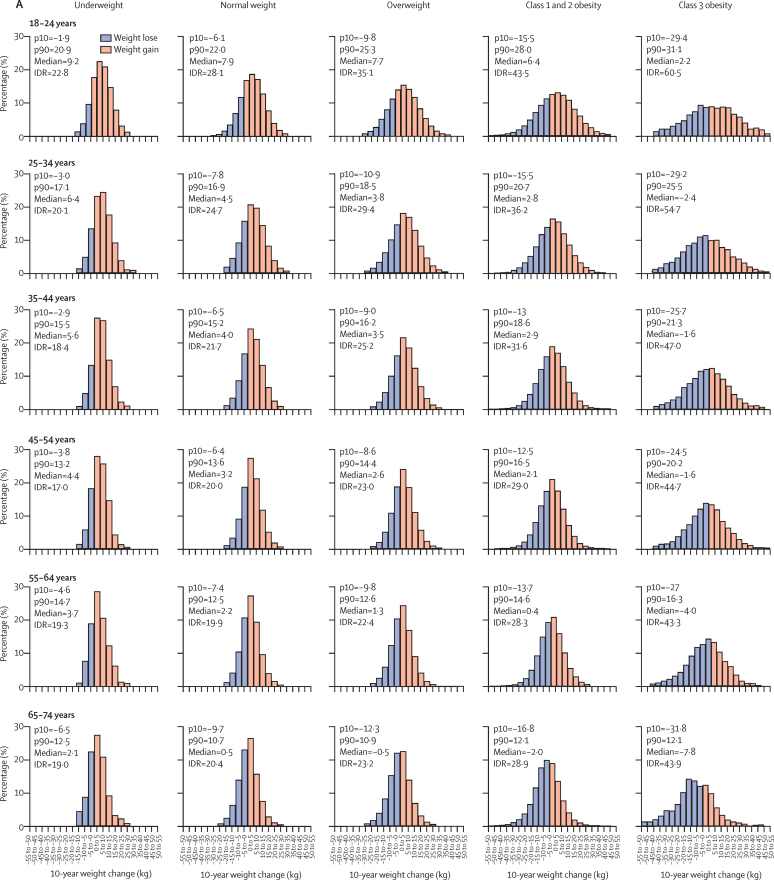

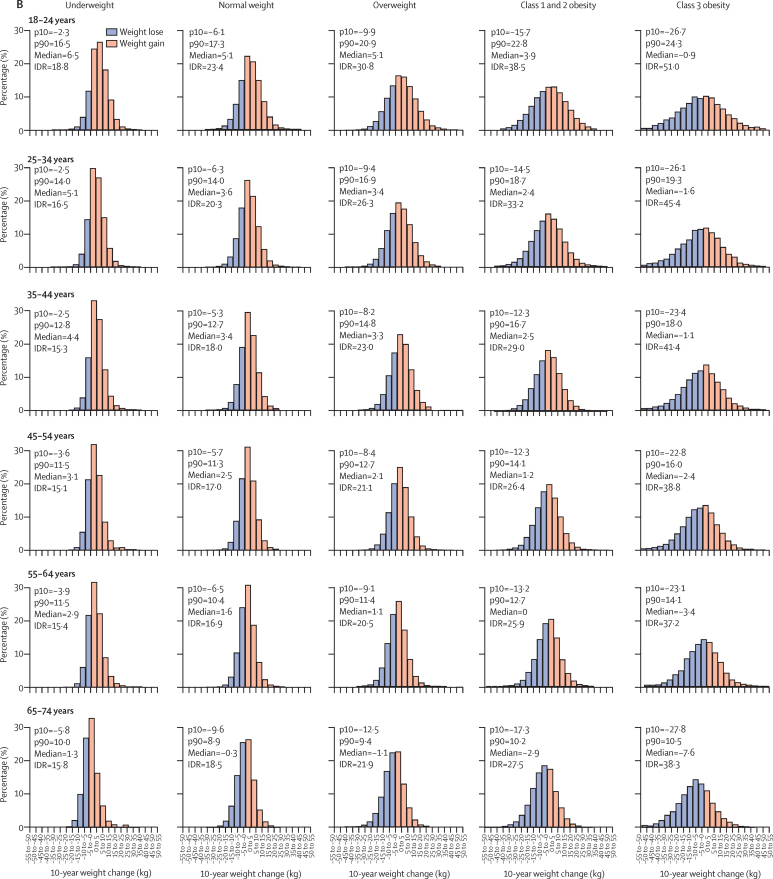
Distribution of weight changes at 10 years in men (A) and women (B) by age and BMI category In A, 470 932 men were included in the analysis; an average height of 1·76 m was assumed. In B, 646 392 women were included in the analysis; an average height of 1·62 m was assumed. IDR=interdecile range. p10=10th percentile. p90=90th percentile.

Adults in the youngest age group (18–24 years) had the highest increase in weight at 10 years, and the widest distribution in each BMI category (figure 2A, B). Each of these measures decreased progressively with increasing age in each BMI category. For example, in men aged 18–24 years in the normal weight BMI category, the median weight change was 7·9 kg and the 90th percentile was 22·0 kg compared with a median weight change of 0·5 kg and a 90th percentile of 10·7 kg in men aged 65–74 years (figure 2A). Similar findings were observed for 1-year and 5-year weight changes in both men and women (appendix p 29).

**Figure 3:**
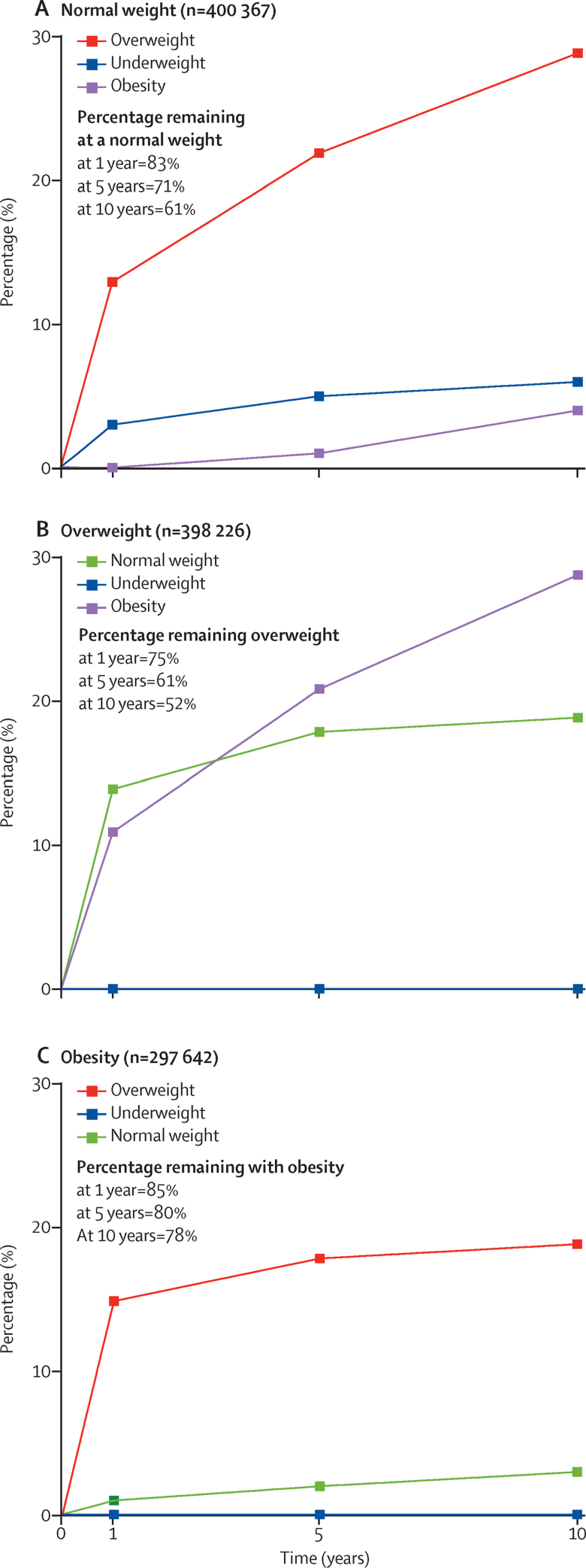
Estimated age-standardised risk of transitioning to normal weight (A), overweight (B), and obesity (C) according to BMI category at 1, 5, and 10 years of follow-up The risk of transitioning was standardised to the English population based on age structure (using information from Office of National Statistics) and prevalence of BMI categories (using data from the Health Survey for England) between 1998 and 2016. For a detailed description of the methods used for this analysis, see the appendix (pp 20–23).

We estimated that, in the normal weight BMI category, 13% transitioned to the overweight category at 1 year, 22% at 5 years, and 29% at 10 years (figure 3). In the overweight BMI category, 11% transitioned to the obesity category at 1 year, 21% at 5 years, and 29% at 10 years. In the obesity BMI category, we estimated that 15% transitioned to the overweight category at 1 year, 18% at 5 years, and 19% at 10 years.

**Figure 4:**
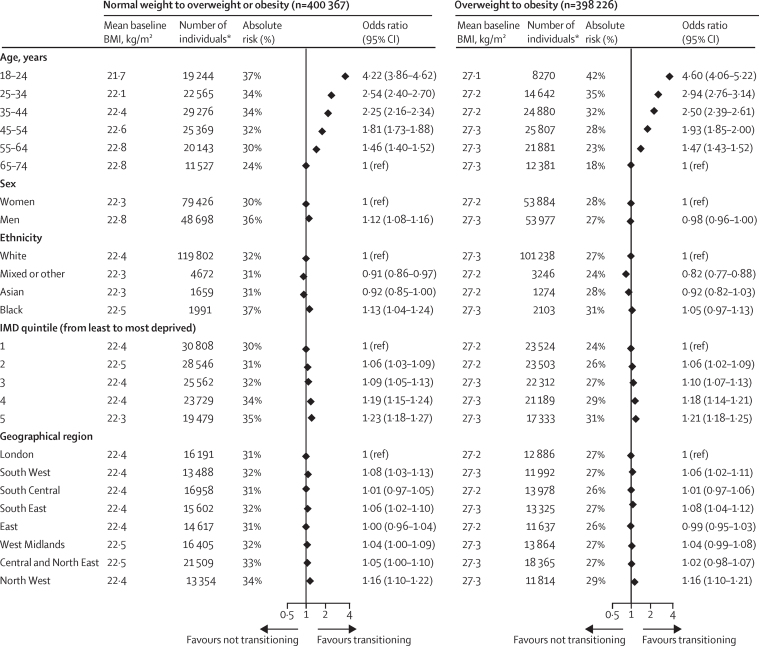

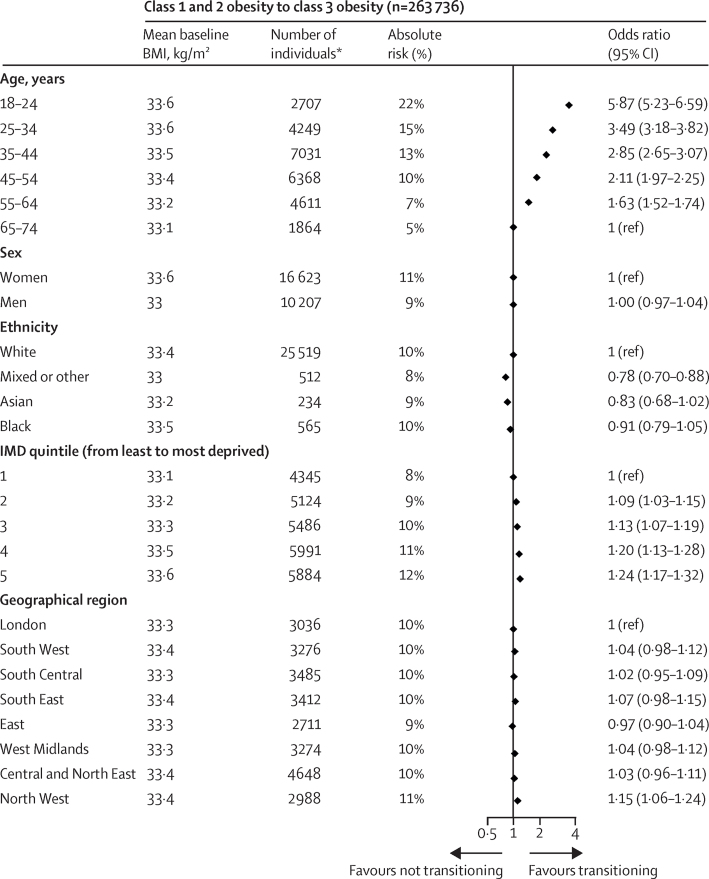
Absolute risk and odds ratio of transitioning from normal weight to overweight or obesity BMI categories, from overweight to obesity BMI categories, and from class 1 and 2 obesity to class 3 obesity BMI categories at 10 years, according to age, sex, ethnicity, degree of social deprivation, and geographical region Odds ratios were mutually adjusted for BMI (at baseline), age group, sex, IMD quintile, ethnicity, geographical region, use of diuretics, and the prevalence of cardiovascular disease, cancer, diabetes, hypertension, mental health disorders (depression, anxiety, stress, phobia, schizophrenia, bipolar disorder, or affective disorder), and other chronic diseases (HIV, chronic obstructive pulmonary disease, neurological disease [dementia], rheumatological disease [rheumatoid arthritis, gout, or systemic lupus erythematosus], gastrointestinal disease (inflammatory bowel disease), and renal disease [chronic kidney disease or renal failure]). IMD=Index of Multiple Deprivation. *Refers to the number of individuals who transitioned to a higher BMI category; for ethnicity and IMD (the two variables with missing values), the numbers were calculated from the first imputation.

Compared with adults in the oldest age group (65–74 years), adults in the youngest age group (18–24 years) had the highest relative risk (OR 4·22 [95% CI 3·86–4·62]) and absolute risk (37% *vs* 24%) of transitioning from the normal weight BMI category to overweight or obesity categories at 10 years (figure 4). Other demographic factors, such as degree of social deprivation (most deprived *vs* least deprived OR 1·23 [1·18–1·27]), sex (men *vs* women OR 1·12 [1·08–1·16]) and ethnicity (Black individuals *vs* White individuals OR 1·13 [1·04–1·24]), were less associated with this transition. Compared with the 65–74 years age group, individuals in the 18–24 years age group had the highest relative risk (OR 4·60 [4·06–5·22]) and absolute risk (42% *vs* 18%) of transitioning from the overweight to the obesity BMI category, and the highest relative risk (OR 5·87 [5·23–6·59]) and absolute risk (22% *vs* 5%) of transitioning from class 1 and 2 obesity to class 3 obesity. We found that the association between IMD and transition to a higher BMI category was even more pronounced among individuals in the 18–24 years age group compared with older age groups (appendix p 30).

We also considered the absolute and relative risks of transitioning from normal weight to underweight BMI categories and for remaining in the obesity BMI category over the 10-year period. Compared with the other age groups, individuals in the 65–74 years age group had a higher risk of transitioning from normal weight to underweight BMI categories (appendix p 31). We also observed that individuals aged 35–54 years had the highest absolute risk (84% in the 35–44 years age group and 83% in the 45–54 years age group) and OR (35–44 years *vs* 65–74 years OR 2·09 [95% CI 1·97–2·22]; 45–54 years *vs* 65–74 years 1·97 [1·87–2·08]) of remaining in the obesity BMI category at 10 years. We observed similar results for the youngest age group for the transitions at higher BMI categories at 1 and 5 years (appendix pp 32–33).

**Figure 5:**
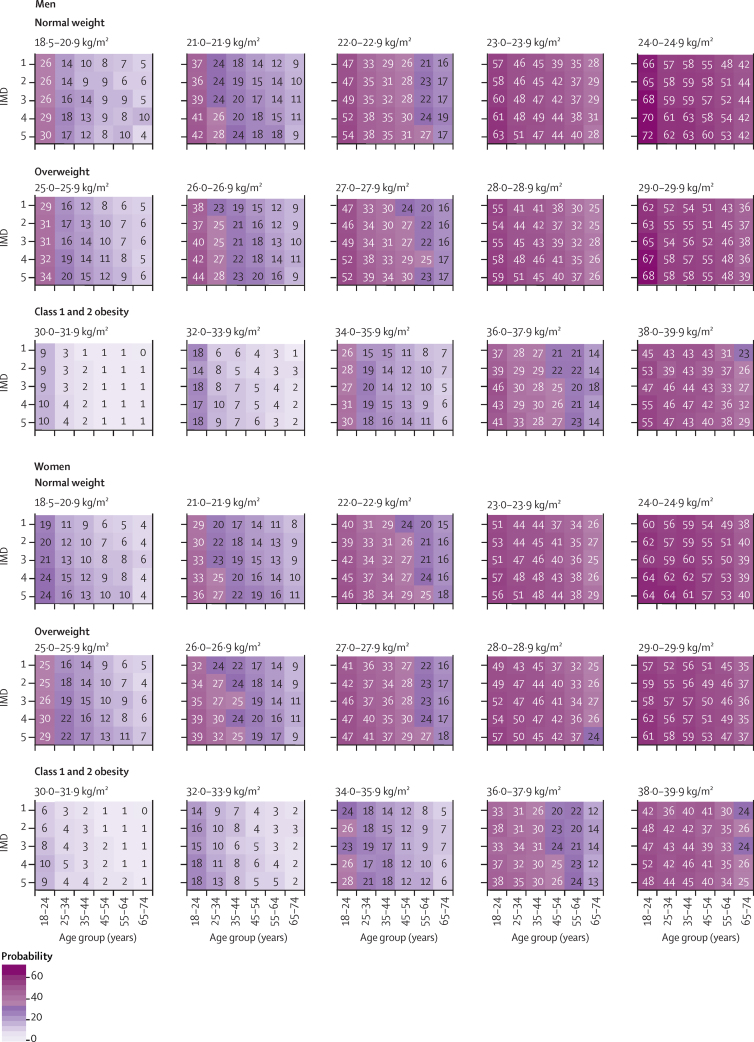
Absolute risk of transitioning to a higher BMI category over 10 years, according to baseline BMI category, age, sex, and IMD quintile This analysis included 1 117 324 individuals; across the 900 strata, there are at least 1000 individuals in 458 (51%) strata, and at least 100 individuals in 890 (99%) strata. IMD=Index of Multiple Deprivation.

We next developed the risk calculator for estimating the risk of transitioning between BMI categories, combining age, sex, degree of social deprivation, and initial BMI. We show that, within each stratum, the risks for transitioning to higher BMI categories were substantially higher in the youngest adult age group than in older age groups. For example, compared with men aged 65–74 years in the third IMD quintile who were close to the BMI transition cutoffs, the risk of those aged 18–24 years transitioning from the normal weight to the overweight BMI category at 10 years was 68% versus 44%, from the overweight to the obesity BMI category was 65% versus 38%, and from the class 1 and 2 obesity to class 3 obesity BMI category was 47% versus 27% (figure 5). By contrast, there were only modest modifications of the influence of the risk of transitioning to higher BMI categories by degree of social deprivation and sex (figure 5; see also the online risk calculator). We found consistent patterns of age-associated transitions to higher BMI categories at 1 year (appendix p 34) and 5 years (appendix p 35).

The sensitivity analyses indicated that our findings were robust when we applied the same analysis: (1) without controlling for the prevalent chronic conditions (appendix p 38); (2) excluding individuals with prevalent chronic diseases at baseline (appendix p 39); (3) excluding individuals with prevalent chronic diseases and those who developed chronic conditions during follow-up (appendix p 40); (4) without accounting for the delta values after the multiple imputation (appendix p 42); and (5) excluding all missing values (complete case analysis; appendix p 41). We also found that the results remained largely the same in individuals who had never smoked, and in former and current smokers (appendix p 43). Compared with individuals who had never smoked, former smokers had a higher risk of transitioning to a higher BMI category, whereas current smokers had a lower risk (appendix p 44).

## Discussion

Young adults (aged 18–24 years) have the highest absolute and relative risk for transitioning to a higher BMI category than older age groups; smaller additional contributions to the risk of BMI changes from living in socially deprived regions and ethnicity were also observed. In the largest study done to date, we provide the first high-resolution obesity risk calculator based on age, sex, degree of social deprivation, ethnicity, and current BMI to identify the risk of intra-individual changes in BMI and transitioning across BMI categories. Our findings show that it is possible to identify individuals at the highest risk of weight gain using EHRs and information readily available to public health agencies, and that young adults should be a major focus of strategies to prevent the onset of overweight and obesity.

We did this large-scale study (involving more than two million individuals) to provide high-resolution estimates of BMI change across 900 strata, defined by age (six groups), sex, social deprivation (five groups), and initial BMI (15 categories). Most of the individual strata contained more than 1000 individuals. Approximately 77% of adults have at least one BMI measurement recorded in their primary health-care record; a percentage that is higher compared with the response rates in consented cross-sectional surveys such as HSE^14^ and NHANES.^15^ For repeated measures, completeness for 1-year BMI changes in CALIBER improved from 47% before 2005 to 53% from 2006 to 2016 in our study. Previous studies have shown the validity of single measures of BMI recorded in primary care,^11^ and of BMI changes in people with obesity^17^ or after bariatric surgery.^18^ Our study replicates (and extends) previous findings from smaller longitudinal studies (appendix pp 3–4) using large-scale EHRs. We propose that the monitoring of population weight data by public health agencies should involve incorporating information on intra-individual weight changes from EHRs, which are becoming increasingly available.^19^, ^20^

Adults in the youngest age group (18–24 years) had the highest absolute and relative risk of transitioning from the normal weight to overweight or obesity BMI categories, from the overweight to obesity BMI categories, and from class 1 and 2 obesity to class 3 obesity BMI categories at 1, 5, and 10 years. Individuals aged 35–54 years had the highest risk for remaining in the obesity BMI category, further emphasising the importance of early intervention.

Our findings have policy implications in three inter-related areas: (1) obesity prevention; (2) the health priorities of young adults; and (3) addressing lockdown-associated weight gain. First, obesity prevention policies should identify groups at a high risk of weight gain for targeted interventions. There could be key opportunities for intervention in young adults because certain factors, such as changes in dietary and exercise behaviours^21^ and relationships,^22^ being in higher education, and employment status,^23^ can influence weight gain. For example, BMI can increase in women and men during the course of cohabiting relationships and marriage.^22^ Identifying groups at the highest risk of weight gain is not currently recommended in obesity prevention policies,^24^ and is not mentioned in reports on new strategies in obesity prevention.^1^ Most of the attention of current obesity prevention guidelines is directed mainly at individuals who already have obesity.^24^ As the evidence presented in our study suggests, the opportunity to modify weight gain is greatest in individuals who are young and do not yet have obesity. Our findings also suggest that regional and national initiatives aimed at socially deprived areas might be complemented by targeting young adults. Second, young adults are increasingly a focus of health policies seeking to address a wide range of issues, including substance abuse, sexual health, mental health, violence and injuries, and criminality.^21^ An extensive review of preventive interventions targeted to young adults made no mention that this group of individuals are at highest risk of weight gain, and found that there were no programmes addressing overweight, obesity, or healthy living.^25^ Additionally, the increasing trends in BMI are combined with an increasing prevalence of diabetes in young adults.^10^ From the perspective of both obesity prevention and health in young adults, it is important to address the challenge that, with the exception of pregnancy complications, most of the adverse health consequences of obesity and weight gain (including severe COVID-19, cardiovascular disease, and type 2 diabetes)^26^ occur decades later, in middle-age and older age. Finally, repeated lockdowns during the COVID-19 pandemic have profoundly reduced the amount of physical activity and energy expenditure in young adults, which has had adverse effects on weight gain.^27^

Population-based EHRs are the only high-resolution source of information on changes in BMI with a sufficient sample size to estimate risk across combinations of age, sex, ethnicity, degree of social deprivation, and BMI category. Our study was the first large-scale study to focus on modern BMI change patterns at 1, 5, and 10 years at a high resolution, taking socioeconomic and demographic factors into consideration.

Our study has important limitations. We focused on BMI changes at 1, 5, and 10 years; however, individuals had their bodyweight measurements taken at their general practice clinic infrequently, and younger (and healthier) individuals had their bodyweight measurements taken less frequently than older indviduals.^11^ To address this problem, we considered time windows within which individuals had a pair of recorded BMI measurements, and assumed linear BMI changes in these time windows. Despite the large sample size of our study, it is possible that our sample was not representative for estimating changes in BMI, as the individuals who had their bodyweight measured multiple times at the general practice clinic had a higher prevalence of chronic disease than those with one or no measurement. A challenge to any study investigating long-term BMI changes is loss to follow-up; the proportion of missing pairs of BMI observations was 48% in the 1-year time window, 52% in the 5-year time window, and 59% in the 10-year time window, comprising individuals who had died or were no longer registered with their general practice. To account for this limitation, we applied multiple imputation with delta-adjustment (to account for missingness not at random)^16^ using mean BMI change data from the HSE. Our results were robust to sensitivity analyses using a complete case analysis (no imputation of missing values) and without delta adjustment. However, we cannot exclude the possibility that we mis-specified the imputation models, even though it is unlikely that the resulting bias would be major. In addition, because we used observational data, it is possible that some of the health variables we used, both in the imputation model and in the analysis presented in figure 4, were prone to misclassification bias. Furthermore, since a small proportion of people aged 18 years had not attained their adult height,^28^ BMI changes in a small proportion of individuals in the youngest age group might have been slightly underestimated. Comparing data from CALIBER and the HSE, mean BMI values according to age, sex, and calendar year were not significantly different in adults aged 18–54 years (ie, the 95% CIs included 0). However, among individuals aged 65–74 years there was some evidence that BMI measurements from the HSE were higher than those from CALIBER (appendix p 7); nevertheless, we included age in the imputation models, and we applied the delta-adjustment to correct any remaining (small) differences at the end.

Our findings suggest the importance of appropriate interventions for obesity prevention at both population and individual levels. At the population level, structural mechanisms (eg, access to housing, affordable healthy food choices, and physical activity opportunities) might influence the risk of transitioning to the obesity BMI category in young adults. There is a need to develop interventions targeted at groups of individuals who have the highest risk of weight gain, with the aim of achieving weight maintenance in the normal weight BMI category. Given the projected weight gain in children and adolescents, complementary approaches before and after the age of 18 years should be followed.^29^ At the individual level, is it possible to communicate information on risk of weight gain, and incorporate this information into interventions designed to affect the knowledge, attitude, and behaviour of young adults? Individuals might have, or seek, additional and actionable predictive information (eg, accelerometery data), which could inform the design of interventions. In addition, to what extent is there a role for primary care professionals^30^ and other clinicians in using and recording risk of weight gain information alongside other actionable information in the EHR?

In summary, we found that young adults aged 18–24 years had the highest risk of transitioning to a higher BMI category compared with older age groups. Compared with age, other sociodemographic factors had weaker absolute and relative associations with weight gain and transitioning to higher BMI categories. Young adulthood offers an important, and currently neglected, opportunity for both population-level prevention of the onset of obesity and the identification of individuals at high risk of weight gain for targeted interventions.

## Data sharing

Individual participant data are available from the CPRD. A data dictionary with details of the definitions of the variables used in the study is available at https://www.caliberresearch.org/portal/codelists. Estimates for weight change can be found via our online tool at bmi.caliberresearch.org.

## Declaration of interests

RLB reports consulting fees from Novo Nordisk, ViiV Healthcare, Pfizer, and Gila Therapeutics; payment or honoraria for lectures, presentations, speakers bureaus, manuscript writing or educational events from Novo Nordisk, ViiV Healthcare, International Medical Press, and Medscape; participation on a data safety monitoring board or advisory board for Novo Nordisk, Pfizer, and ViiV Healthcare; being a committee member of the British Obesity and Metabolic Surgery Society, a trustee for the Association for the Study of Obesity, a scientific chair of the International Federation for the Surgery for Obesity (IFSO) and metabolic disorders European Chapter, a chair of the Royal College of Physicians advisory Committee on Nutrition, Weight and Health, an European Society of Endocrinology clinical committee member, and a trustee of Obesity Empowerment Network UK; being a member of the IFSO scientific committee; being a member of the NICE Weight Management Guideline Development Group; and being a principal investigator on two obesity clinical trials of cagrilintide versus placebo and semaglutide versus placebo (sponsored by Novo Nordisk), and one clinical trial of liraglutide versus placebo (both drugs were provided by Novo Nordisk). AB reports grants from Astra Zeneca. All other authors declare no competing interests.
