# Supplementary material for: Identifying adults at high-risk for change in weight and BMI in England: a longitudinal, large-scale, population-based cohort study using electronic health records
Source: Lancet Diabetes Endocrinol. 2021 Oct;9(10):681–94. doi: 10.1016/S2213-8587(21)00207-2 (PMC8440227; doi:10.1016/S2213-8587(21)00207-2)
Supplement: Supplementary appendix [file mmc1.pdf]

THE LANCET
Diabetes & Endocrinology

Supplementary appendix

This appendix formed part of the original submission and has been peer reviewed. We post it as supplied by the authors.

Supplement to: Katsoulis M, Lai G A, Diaz-Ordaz K, et al. Identifying adults at high-risk for change in weight and BMI in England: a longitudinal, large-scale, population-based cohort study using electronic health records. *Lancet Diabetes Endocrinol* 2021; published online September 2. http://dx.doi.org/10.1016/S2213-8587(21)00239-4.

Appendix

Identifying adults at high-risk for change in weight and body mass index: a large-scale population-based cohort using Electronic Health Records

Contents

Section 1

Evidence before this study from population-based cohorts of within person BMI change page 3-4

Display Items: Table S1

Section 2

Comparison of CPRD (electronic health records) versus other datasets page 5-8

Display Items: Figure S1-S3

Section 3: Statistical methods & details on the data used

3.1: Variables used in this analysis page 9

3.2: Calculation of the 1-, 5- and 10-year BMI change using window periods page 10-12

Display Items: Tables S2 – S3; Figure S4

3.3: Multiple imputation of BMI change under MNAR page 13-19

Display Items: Tables S4 – S10

3.4: Calculation of age-standardised transitions between BMI groups page 20-23

Display Items: Tables S11 – S14

3.5: Calculating the odds ratios of the sociodemographic factors for the transition between BMI category within 1-, 5- and 10-year periods page 24

3.6: Converting an odds ratio to a relative risk (for the online calculator) page 25

Section 4

Additional results page 26-44

Display Items: Tables S15 – S17; Figures S5 – S18

Section 1: Evidence before this study from population-based cohorts of within person BMI change

Table S1: Cohort studies of within-individual BMI change in adults in relation to demographic factors, with at least 5,000 participants and at least 1,000 young adults (18-24 years old), sorted by their sample size

Author, publication year	Total N	N of young adults aged 18-24y†	BMI meas. after 2010	Population based	EHR	Duration of follow up<br>1yr, 5yrs, 10yrs	Transitions between BMI categories<br>Normal weight to overweight / overweight to obesity / obesity to severe obesity	Age range at baseline (years)	Additional demographic factors considered beyond age and sex?	
									Socioeconomic status	Ethnicity
**Our Study**	2 M	200K	●	●	●	●●●	●●●	18-74	●	●
Peter RS et al, 2014 [1]	185K	15K	●	●	○	○●○	○○○	20-85	○	○
Fildes et al. 2015 [2]	166K	10K	●	○	●	○○●	○○○	≥20	○	○
Liu et al [3]	60K	8K	●	●	○	○●○	○○○	18-70	○	●
Droyvold et al, 2006 [4]	45K	1.5K	○	●	○	○○●	○○○	≥20	○	○
Tirosh et al[5]	38K	38K	○	○	○	○●●	○○○	≥17	○	○
Coogan PE et al, 2012 [6]	23K	5K	○	○	○	○○○	○○○	21-55	●	●
Ouyang et al, 2015 [7]	18K	3K	●	●	○	○●●	○○○	18-60	●	○
Lebenbaum et al, 2018 [8]	18K	3K	●	●	○	○○●	○●○	≥20	○	●
Holowko et al [8]	14K	14K	○	○	○	○○○	○○○	18-23	●	●
Paynter L. et al, 2015 [10]	12K	2K	○	●	○	○●○	○○○	18-66	○	○
Wilsgaard et al, 2005 [11]	11K	1K	○	○	○	○●●	○○○	20-61	●	○
Cllarke et al 2009 [12]	11K	1K	○	○	○	○●●	○○○	18-45	●	●
Malhatra et al 2013 [13]	10K	5K	○	○	○	○●●	○○○	14-22	●	●
Haheim et al, 2006 [14]	7K	1K	○	○	○	○○○	○○○	20-49	○	○
Setia MS et al, 2009 [15]	5.5K	1K	○	●	○	●●●	○○○	18-54	○	●
Caman OK et al, 2013 [16]	5.5K	1K	○	●	○	○●●	○○○	16-71	○	○
Lewis CE et al, 2000 [17]	5.1K	2.5K	○	○	○	○○●	○○○	18-32	○	●
Dutton et al, 2016 [18]	5K	2.5K	●	○	○	○●●	○○○	18-55	○	●

● feature present
○ feature absent

†Estimation

References

1. Peter RS, Fromm E, Klenk J, Concin H, Nagel G. Change in height, weight, and body mass index: longitudinal data from Austria. *Am J Hum Biol*. 2014;26(5):690-696. doi:10.1002/ajhb.22582.
2. Fildes A, Charlton J, Rudisill C, Littlejohns P, Prevost AT, Gulliford MC. Probability of an Obese Person Attaining Normal Body Weight: Cohort Study Using Electronic Health Records. Am J Public Health. 2015 Sep;105(9):e54-9. doi: 10.2105/AJPH.2015.302773. Epub 2015 Jul 16. PMID: 26180980; PMCID: PMC4539812
3. Liu N, Birstler J, Venkatesh M, Hanrahan LP, Chen G, Funk LM. Weight Loss for Patients With Obesity: An Analysis of Long-Term Electronic Health Record Data. *Med Care*. 2020;58(3):265-272. doi:10.1097/MLR.0000000000001277
4. Drøyvold WB, Nilsen TI, Krüger O, et al. Change in height, weight and body mass index: Longitudinal data from the HUNT Study in Norway. *Int J Obes (Lond)*. 2006;30(6):935-939. doi:10.1038/sj.ijo.0803178
5. Tirosh A, Shai I, Afek A, Dubnov-Raz G, Ayalon N, Gordon B, Derazne E, Tzur D, Shamis A, Vinker S, Rudich A. Adolescent BMI trajectory and risk of diabetes versus coronary disease. N Engl J Med. 2011;364(14):1315-25
6. Coogan PE, Wise LA, Cozier YC, Palmer JR, Rosenberg L. Lifecourse educational status in relation to weight gain in African American women. *Ethn Dis*. 2012;22(2):198-206.
7. Ouyang Y, Wang H, Su C, et al. Use of quantile regression to investigate changes in the body mass index distribution of Chinese adults aged 18-60 years: a longitudinal study. BMC Public Health. 2015;15:278. Published 2015 Mar 21. doi:10.1186/s12889-015-1606-8
8. Lebenbaum M, Espin-Garcia O, Li Y, Rosella LC. Development and validation of a population based risk algorithm for obesity: The Obesity Population Risk Tool (OPoRT). PLoS One. 2018;13(1):e0191169
9. Holowko N, Jones M, Tooth L, Koupil I, Mishra G. Educational mobility and weight gain over 13 years in a longitudinal study of young women. *BMC Public Health*. 2014;14:1219. Published 2014 Nov 25. doi:10.1186/1471-2458-14-1219
10. Paynter L, Koehler E, Howard AG, Herring AH, Gordon-Larsen P. Characterizing long-term patterns of weight change in China using latent class trajectory modeling. *PLoS One*. 2015;10(2):e0116190
11. Wilsgaard T, Jacobsen BK, Arnesen E. Determining lifestyle correlates of body mass index using multilevel analyses: the Tromsø Study, 1979-2001. *Am J Epidemiol*. 2005;162(12):1179-1188. doi:10.1093/aje/kwi328
12. Clarke P, O'Malley PM, Johnston LD, Schulenberg JE. Social disparities in BMI trajectories across adulthood by gender, race/ethnicity and lifetime socio-economic position: 1986-2004. Int J Epidemiol. 2009;38(2):499-509. doi:10.1093/ije/dyn214
13. Malhotra R, Ostbye T, Riley CM, Finkelstein EA. Young adult weight trajectories through midlife by body mass category [published correction appears in Obesity (Silver Spring). 2014 Jul;22(7):1770]. *Obesity (Silver Spring)*. 2013;21(9):1923-1934. doi:10.1002/oby.20318
14. Lund Haheim L, Lund Larsen PG, Sogaard AJ, Holme I. Risk factors associated with body mass index increase in men at 28 years follow-up. *QJM*. 2006;99(10):665-671. doi:10.1093/qjmed/hcl090
15. Setia MS, Quesnel-Vallee A, Abrahamowicz M, Tousignant P, Lynch J. Convergence of body mass index of immigrants to the Canadian-born population: evidence from the National Population Health Survey (1994-2006). *Eur J Epidemiol*. 2009;24(10):611-623. doi:10.1007/s10654-009-9373-4
16. Caman OK, Calling S, Midlöv P, Sundquist J, Sundquist K, Johansson SE. Longitudinal age-and cohort trends in body mass index in Sweden--a 24-year follow-up study. BMC Public Health. 2013;13:893.
17. Lewis CE, Jacobs DR Jr, McCreath H, et al. Weight gain continues in the 1990s: 10-year trends in weight and overweight from the CARDIA study. Coronary Artery Risk Development in Young Adults. *Am J Epidemiol*. 2000;151(12):1172-1181. doi:10.1093/oxfordjournals.aje.a010167
18. Dutton, G.R., Kim, Y., Jacobs, D.R., Jr., Li, X., Loria, C.M., Reis, J.P., Carnethon, M., Durant, N.H., Gordon-Larsen, P., Shikany, J.M., Sidney, S. and Lewis, C.E. (2016), 25-year weight gain in a racially balanced sample of U .S . adults: The CARDIA study. Obesity, 24: 1962-1968. doi:10.1002/oby.21573

Section 2: Comparison of CPRD (electronic health records) versus other datasets

Figure S1: Estimates of mean BMI levels in England between 1998 and 2016 from Electronic Health Records (EHR; 2M individuals) and Health Survey from England (HSE: 150K), by age and sex

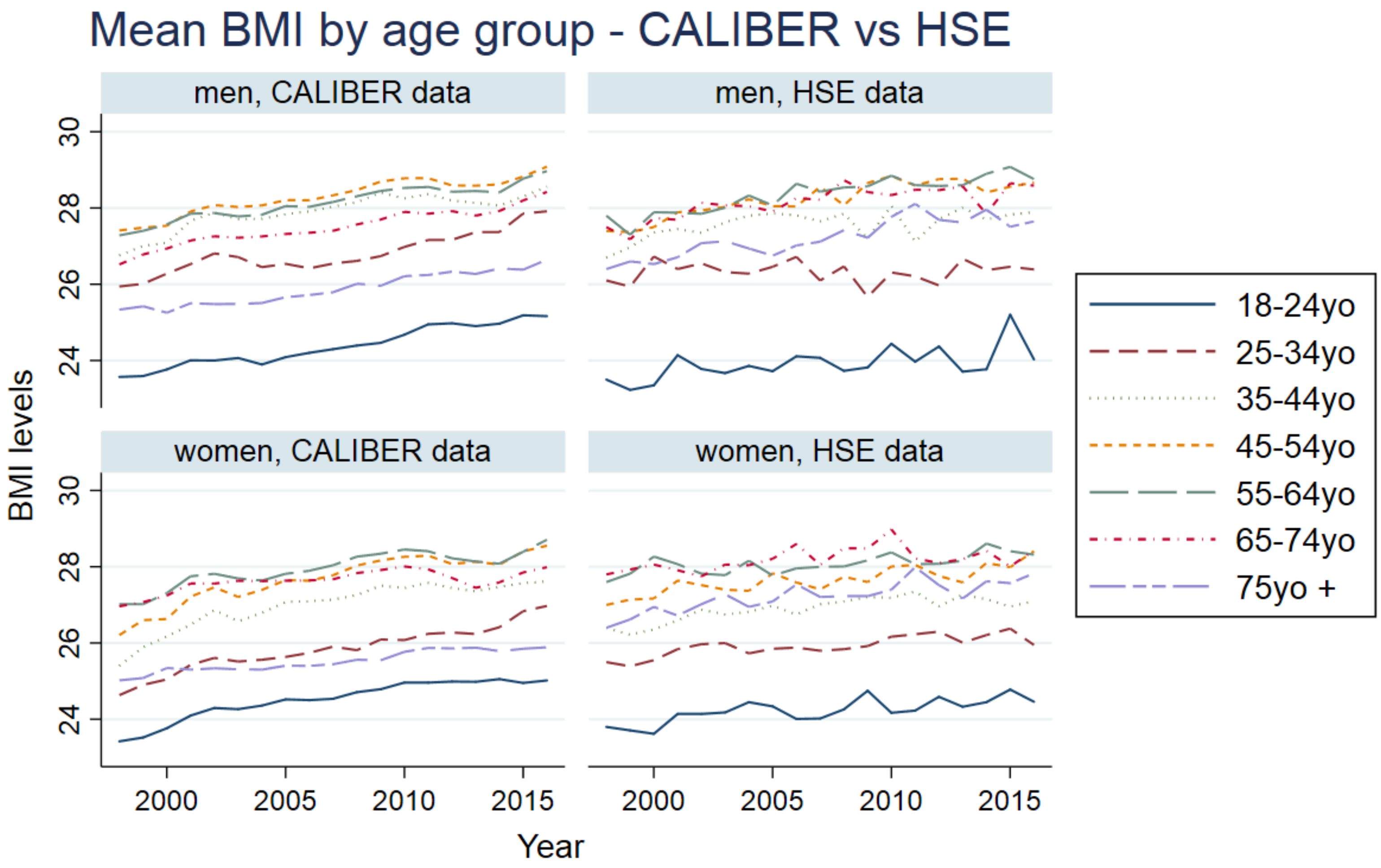

Figure S2: Estimates of the mean BMI differences in England between 1998 and 2016 from Electronic Health Records (EHR; 2M individuals) and Health Survey from England (HSE: 150K), by age and sex

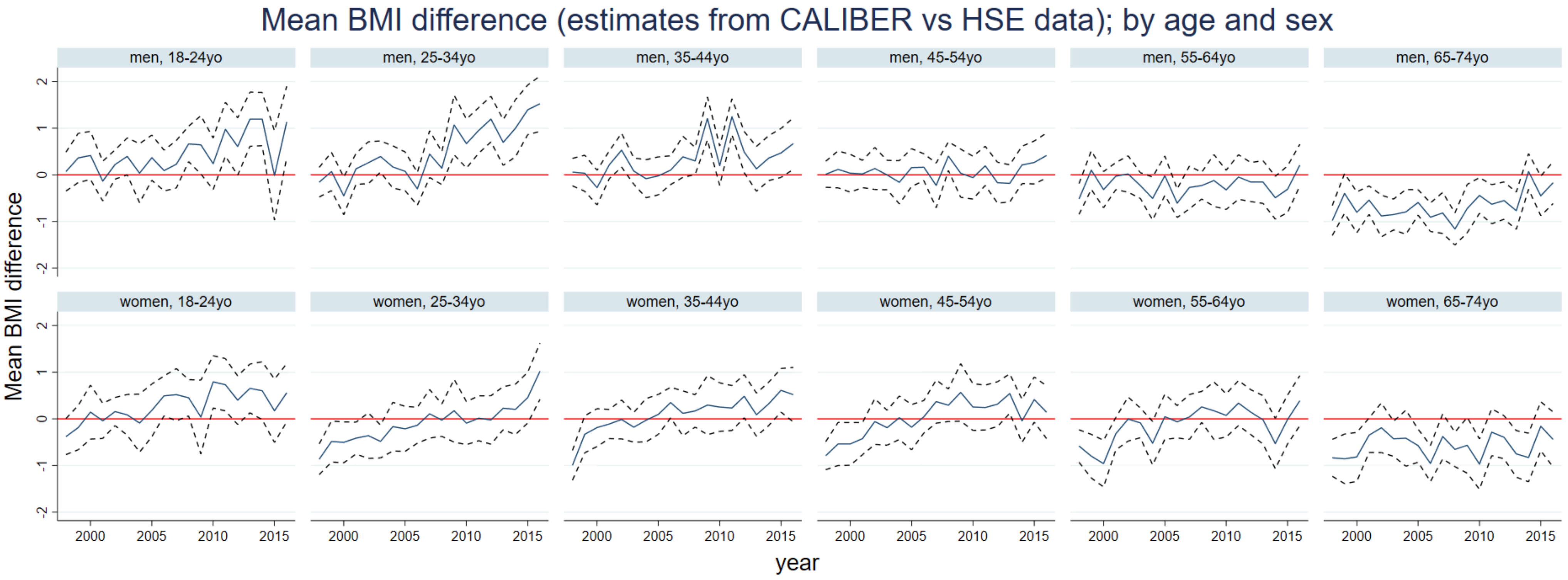

Figure S3: Trends in mean BMI 1999-2016 by age in England (EHR data from CALIBER, n=2M) and the US (survey data NHANES n=47K)

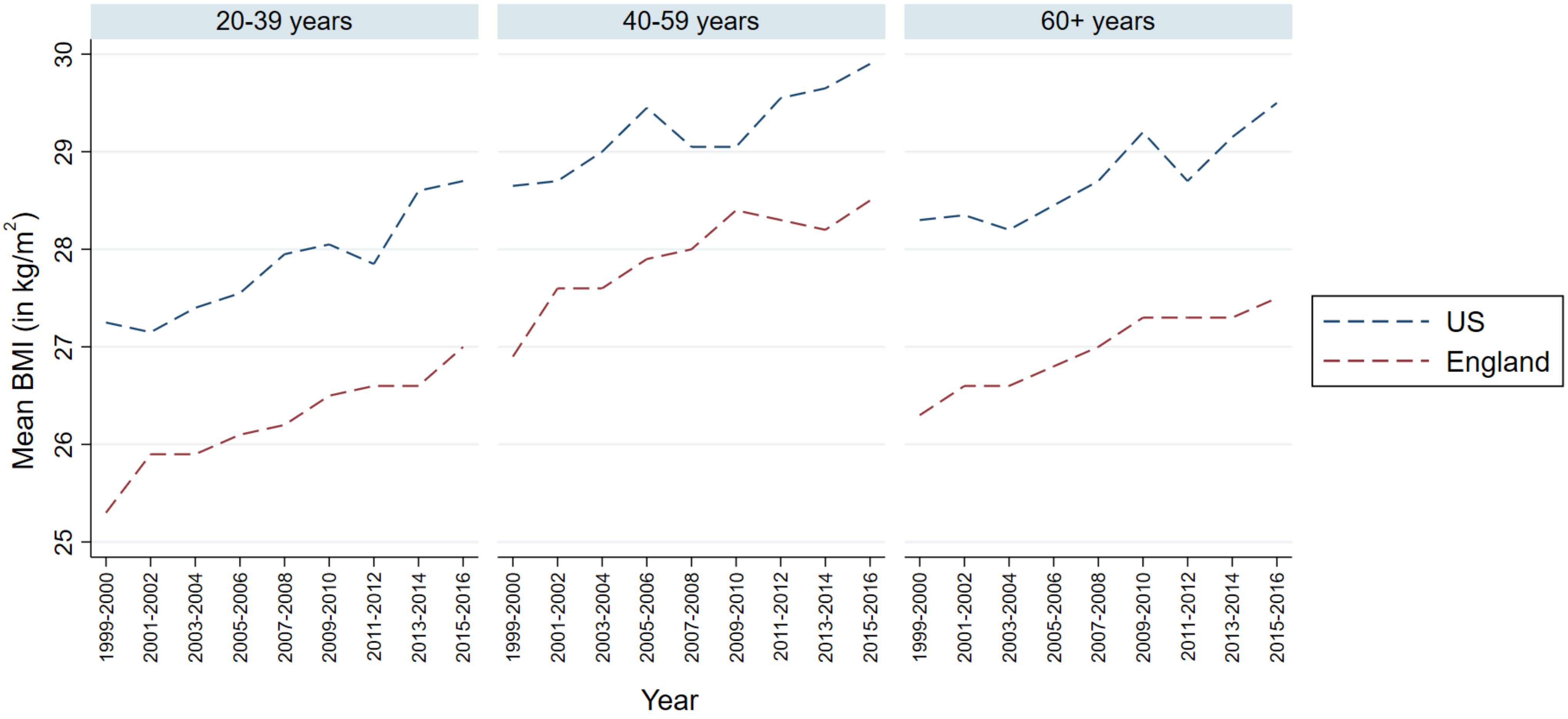

Section 3: Statistical methods

3.1. Variables used for imputation models and analysis

In this paper, we used the following variables in the imputation models and the analysis models.

Sociodemographic: Age, sex, ethnicity, index of multiple deprivation, region

Lifestyles: Smoking status and physical activity

Cardiovascular diseases (information used from both primary care and secondary care): Stable and unstable angina, myocardial infarction, ischaemic and haemorrhagic stroke, abdominal aortic aneurism, peripheral arterial disease, coronary heart disease, heart failure, transient ischemic attack

Cancer apart from non-melanoma skin cancer

Mental health disorders (information used from both primary care and secondary care): depression, anxiety, stress, phobia, schizophrenia, bipolar disorder, affective disorder

Other chronic diseases (information used from both primary care and secondary care): hypertension, diabetes, HIV, chronic obstructive pulmonary disease, neurological (dementia), rheumatological (rheumatoid arthritis, gout, systemic lupus erythematosus), gastro-intestinal (inflammatory bowel disease) and renal (chronic kidney disease, renal failure)

Medication: Diuretics

Other variables: Bariatric surgery, Family history of CVD

3.2. Calculation of the 1-, 5- and 10-year BMI change using window periods

To estimate the 1-, 5- and 10-year BMI change periods, we used window periods (6 months-2 years, 4 years-6 years and 8 years-12 years respectively) in which individuals had at least 2 BMI measurements (see Table S2).

Table S2: Window periods used to select a pair of BMI observations and estimate BMI change

Estimated Period	Window period
1-year BMI change	6 months to 2 years
5-year BMI change	4 years to 6 years
10-year BMI change	8 years to 12 years

If an individual had more than one pair of BMI measurements, we selected at random one of them. To estimate the 1-, 5- and 10-year BMI change, we assume that BMI change is linear in this window, i.e. if an individual's BMI decreased by 2.6 kg/m^2 in 1.3 years, we assume that the 1-year BMI change for this individual in -2 kg/m^2.

Below, we present an example of a hypothetical individual, her BMI measurements between 1998 and 2016 and we explain how her BMI measurements contribute in the calculation of the 1-, 5- and 10-year BMI change. Her BMI was measured at 27.2 kg/m^2 in 23/11/2001, at 27.2 kg/m^2 in 8/10/2002, at 27.2 kg/m^2 in 2/4/2003 and at 27.2 kg/m^2 in 28/3/2009 (see below in Figure S4).

Figure S4: Estimating BMI change for a hypothetic individual

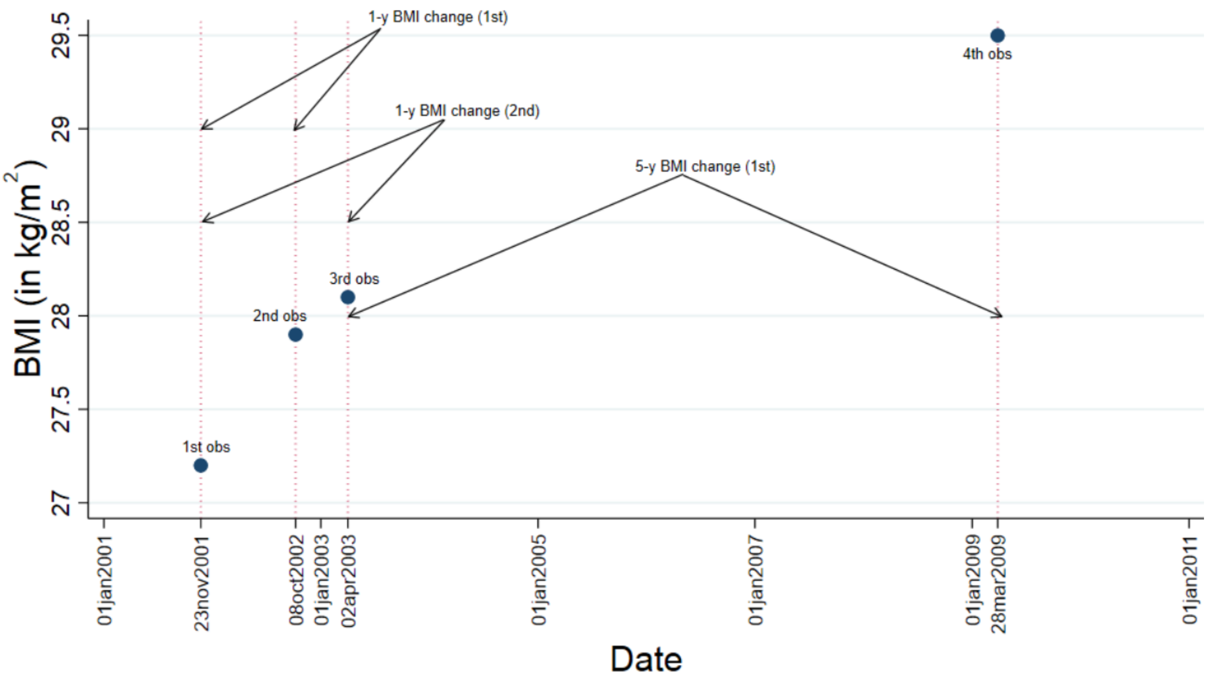

From her data, the pairs of BMI observation that contribute to the 1-year BMI change (i.e. within 6 months to 2 years) are

1. (1st, 2nd) BMI measurements
2. (1st, 3rd) BMI measurements

The pairs of BMI observation that contribute to the 5-year BMI change (i.e. within 4 to 6 years) are

1. (3rd, 4th) BMI measurements

Additionally, she has no pairs of BMI measurements which contribute to the 5-year BMI change (i.e. within 8 to 12 years) are

To estimate her contribution to the 1-, 5- and 10-year BMI change, we select at random one pair of BMI observations for each period. So, for example, we select at random her 2nd pair of BMI measurements, i.e. (1st, 3rd) observations. For the calculation of the 5-year BMI change, we have only 1 pair of BMI measurements, i.e. (3rd, 4th) observations.

Moreover, we assumed linear trend within each period, so her contribution to the 1-year BMI change is 0.18% and to the 5-year BMI change is 0.32% (see Table S3). Finally, she will have a missing value for the calculation of the 10-year BMI change.

Table S3: Estimation of BMI change of a hypothetical individual (from Figure S5)

Estimated Period	Pairs of BMI observations contributing	Pair selected	Period between the two BMI measurements	Observed BMI change	Estimated BMI change
1-year BMI change	(1st, 2nd), (1st, 3rd)	(1st, 3rd)	1.36 years	0.25%	0.25% / 1.36 = **0.18%**
5-year BMI change	(3rd, 4th)	(3rd, 4th)	5.98 years	0.38%	5* (0.38% /5.98) = **0.32%**
10-year BMI change	-	-	-	-	-

3.3 Multiple imputation of BMI change under MNAR

In the analysis of 1-, 5-, 10-year BMI change, there were many individuals that had no pair of BMI measurements within the corresponding window period, so these individuals had a missing value for BMI change. For example, the hypothetical individual in Figure S4, she had no pair of BMI measurements between 8 and 12 years, thus resulting without an estimate for the 10-year BMI change.

To tackle the problem of missing values in our analysis, we assumed that the missingness mechanism for BMI change was missing not at random (MNAR). In other words, missing values on BMI change might depend on BMI change itself, as it is more likely that those individuals who don't weigh in frequently in their practice, are healthy and their weight/BMI has not changed. For this reason, we applied multiple imputation with delta adjustment, which is a 2-part method: in the 1st part, we apply multiple imputation for BMI change (and for other covariates), as if missing at random (MAR) held, but then, in the 2nd part, we added delta values to our imputed datasets of BMI change. To account for the fact that some individuals had multiple contacts with the health system, and multiple measurements, we considered a summary variable that recorded the number of visits recorded. More specifically,

1. We created 10 copies of datasets in which missing values of BMI change, ethnicity, index of multiple deprivation (IMD), smoking status and physical activity were replaced by imputed values sampled from their predictive distribution, through multiple imputation by chained equations, which was applied separately in 6 age groups (18-24 years old, 25-34 years old, 35-44 years old, 45-54 years old, 55-64 years old and 65-74 years old). The imputation model in each age group included:

a) Use of diuretics, history of bariatric surgery, prevalence of hypertension, cancer (apart from non-melanoma skin cancer), cardiovascular disease, diabetes, other chronic diseases (dementia, chronic kidney disease, systemic lupus erythematosus, rheumatoid arthritis, gout, ulcerative colitis, Parkinson disease, multiple sclerosis, renal disease, renal failure) and psychological conditions (depression, anxiety, stress, phobia, schizophrenia, bipolar disorder, affective disorder).
b) For each of the 1-, 5- and 10-year period, we additionally considered the use of diuretics and the performance of bariatric surgery during these periods, as well as the presence of all the incident chronic conditions described in a) above,
c) We included ethnicity, index of multiple deprivation, physical activity and smoking status that also had missing values. We considered physical activity and smoking status after 1 or 5 or 10 years after the BMI measurement, depending on the imputation of the corresponding BMI change
d) We also included age, quadratic age, cubic age, time (from 1998 to year of baseline), quadratic time (from 1998 to year of baseline), family history of CVD
e) BMI at baseline in each BMI category [5 coefficients were created, each for underweight, normal weight, overweight, non-severe obesity (BMI≥30 & BMI<40kg/m^2) and severe obesity (BMI≥40 kg/m^2)]
f) We added one variable for the frequency of contacts of an individual with the health care system during the corresponding period (i.e. one, five and ten years). More specifically, this variable had 6 categories and was modelled as ordered (1: 1 time, 2: 2-3 times, 3: 4-5 times, 4: 6-11 times, 5: 12-23 times and

6: 24 or more times). We additionally added one variable for the frequency of weight measurements in the primary care during the corresponding period (i.e. one, five and ten years). More specifically, this variable had 6 categories and was modelled as ordered (1: 1 time, 2: 2 times, 3: 3 or more times)

We opted not to include seasonality in the imputation models to make them less burdensome computationally. We ended up to this decision because any improvement in the accuracy of the models would be negligible, because when we regressed seasonality (0 if months=January, February, March, October, November, December and 1 otherwise; i.e. the first and the last 3 months of a year vs other "warmer" months) on BMI levels, adjusted for year, age, square age, and cubic age, the association was not significant (see Table S4 below).

For more details, see https://github.com/mkatsoulis82/Identifying-adults-at-high-risk-for-change-in-weight-and-BMI/blob/main/Multiple%20Imputation

Table S4: Association of BMI levels with age, year and seasonality

Factors	beta	95% CI	p-value
**Age (in years)**	0.32	(0.31, 0.33)	<0.001
**Age^2**	-0.025	(-0.027, -0.023)	<0.001
**Age^3**	$-4.9*10^{-6}$	$(-5.9*10^{-6}, 3.9*10^{-6})$	<0.001
**Year**	0.76	(0.74, 0.78)	<0.001
**Seasonality***	0.0075	(-0.0071, 0.0220)	0.315

*Seasonality: 0 if months=January, February, March, October, November, December and 1 otherwise; i.e. the first and the last 3 months of a year vs other "warmer" months

2. We then added delta values to our imputed datasets of BMI change, to address the problem of the missing not at random mechanism. The delta values were derived as follows: We used the estimations of BMI levels from both men and women from the Health Survey from England between 1998 and 2016 for the following age groups: 16-24, 25-34, 35-44, 45-54, 55-64, 65-74 and 75+ years old. We then calculated the

10-year BMI change for each age group, for each year separately. For example, the average 10-year BMI change in men aged 25-34 in 1998, noted $\overline{BMI\_ch25\_34}$, was

$$\overline{BMI\_ch25\_34(1998)} = \frac{\left(\overline{BMI25\_34(2008)} - \overline{BMI25\_34(1998)}\right)}{\overline{BMI25\_34(1998)}} = \frac{27.8 - 26.1}{26.1} = 6.5\%$$

In Table S5, we show which BMI estimates we combine to calculate the 10-year BMI changes for men aged 25-34, from HSE, for all the years from 1998 to 2006. The same procedure was followed for all age groups in men in Table S5, as well as in women in Table S6

Table S5: BMI levels of men estimated from the Health Survey for England (HSE).

**Year**	**Age 16-24**	**Age 25-34**	**Age 35-44**	**Age 45-54**	**Age 55-64**	**Age 65-74**	**Age 75+**
**1998**	23.5	26.1	26.7	27.4	27.8	27.5	26.4
**1999**	23.2	25.9	27.0	27.4	27.3	27.2	26.6
**2000**	23.4	26.7	27.4	27.5	27.9	27.7	26.5
**2001**	24.1	26.4	27.4	27.9	27.9	27.7	26.7
**2002**	23.8	26.6	27.3	27.9	27.9	28.1	27.1
**2003**	23.7	26.3	27.6	28.0	28.0	28.1	27.1
**2004**	23.9	26.3	27.8	28.2	28.3	28.0	26.9
**2005**	23.7	26.5	27.9	28.0	28.1	27.9	26.8
**2006**	24.1	26.7	27.8	28.0	28.6	28.3	27.0
**2007**	24.1	26.1	27.7	28.6	28.4	28.2	27.1
**2008**	23.7	26.5	27.8	28.1	28.5	28.7	27.4
**2009**	23.8	25.7	27.2	28.7	28.6	28.4	27.2
**2010**	24.4	26.3	28.1	28.8	28.9	28.3	27.8
**2011**	24.0	26.2	27.1	28.6	28.6	28.5	28.1
**2012**	24.4	26.0	27.7	28.8	28.6	28.5	27.7
**2013**	23.7	26.7	28.0	28.8	28.6	28.6	27.6
**2014**	23.8	26.4	27.7	28.4	28.9	27.9	28.0
**2015**	25.2	26.5	27.8	28.6	29.1	28.7	27.5
**2016**	24.0	26.4	27.9	28.7	28.8	28.6	27.6

From the circles connected by an arrow, we can derive the average 10-year BMI change for men aged 25-34, for all the years from 1998-2006

Table S6: BMI levels of women estimated from the Health Survey for England (HSE).

Year	Age 16-24	Age 25-34	Age 35-44	Age 45-54	Age 55-64	Age 65-74	Age 75+
**1998**	23.8	25.5	26.4	27.0	27.6	27.8	26.4
**1999**	23.7	25.4	26.2	27.1	27.8	27.9	26.6
**2000**	23.6	25.6	26.4	27.2	28.3	28.1	26.9
**2001**	24.1	25.8	26.6	27.6	28.1	27.9	26.7
**2002**	24.1	26.0	26.9	27.5	27.8	27.7	27.0
**2003**	24.2	26.0	26.7	27.4	27.8	28.1	27.3
**2004**	24.4	25.7	26.8	27.4	28.2	28.0	26.9
**2005**	24.3	25.8	27.0	27.8	27.8	28.2	27.1
**2006**	24.0	25.9	26.8	27.6	28.0	28.6	27.5
**2007**	24.0	25.8	27.0	27.4	28.0	28.0	27.2
**2008**	24.3	25.8	27.1	27.7	28.0	28.5	27.2
**2009**	24.8	25.9	27.2	27.6	28.2	28.5	27.2
**2010**	24.2	26.2	27.2	28.0	28.4	29.0	27.4
**2011**	24.2	26.2	27.4	28.0	28.1	28.2	28.0
**2012**	24.6	26.3	27.0	27.8	28.1	28.1	27.5
**2013**	24.3	26.0	27.3	27.6	28.2	28.2	27.2
**2014**	24.4	26.2	27.2	28.1	28.6	28.4	27.6
**2015**	24.8	26.4	27.0	28.0	28.4	28.0	27.6
**2016**	24.5	25.9	27.1	28.4	28.3	28.4	27.8

We then calculated the average of all the BMI changes between 1998 and 2006 for all age groups, in men and women separately. Of note, we could not calculate BMI change for those individuals aged ≥75 years old. The values for average 10-year BMI change values are presented below, in Table S7.

Table S7: Average 10-year BMI change (%) values for both men and women, estimated from the Health Survey for England (HSE).

Sex	Age 16-24	Age 25-34	Age 35-44	Age 45-54	Age 55-64	Age 65-74
**Men**	10.9%	5.0%	4.2%	3.2%	1.7%	-0.1%
**Women**	8.6%	5.4%	4.8%	3.1%	1.6%	-0.2%

Moreover, we consider all the average 10-year BMI change calculated by the HSE as the reference BMI changes that should match with the ones from CALIBER. For this reason, we name the following variables:

$BMI\_ch\_ref_1$ → *Average BMI change in men aged between 16-24yo*

R_1 → *Observed values of BMI change in men aged between 16-24yo in CALIBER*

***BMI_ch_ref*$_2$** → *Average BMI change in men aged between 25-34yo*

***R*$_2$** → *Observed values of BMI change in men aged between 25-34yo in CALIBER*

…

***BMI_ch_ref*$_6$** → *Average BMI change in men aged between 65-74yo*

***R*$_6$** → *Observed values of BMI change in men aged between 65-74yo in CALIBER*

and we continue with the women, i.e.

***BMI_ch_ref*$_7$** → *Average BMI change in women aged between 16-24yo*

***R*$_7$** → *Observed values of BMI change in women aged between 16-24yo in CALIBER*

***BMI_ch_ref*$_8$** → *Average BMI change in women aged between 25-34yo*

***R*$_8$** → *Observed values of BMI change in women aged between 25-34yo in CALIBER*

…

***BMI_ch_ref*$_{12}$** → *Average BMI change in women aged between 65-74yo*

***R*$_{12}$** → *Observed values of BMI change in women aged between 65-74yo in CALIBER*

We also set **BMI_ch$_1$ – BMI_ch$_{12}$** the estimated BMI change per age group and sex, **n_obs$_1$ - n_obs$_{12}$** the number of individuals with observed BMI change values per age group and sex in CALIBER, as well as **n_mis$_1$ - n_mis$_{12}$** the number of individuals with missing values on BMI change per age group and sex in CALIBER.

We then require that the average 10-year BMI estimates per age group and sex from CALIBER, after multiple imputation, would be the same with the corresponding estimates from HSE. In other words, for each i=1,2,…,12, we set

$$\frac{n\_obs_i * (\overline{BMI\_ch_i * R_i}) + n\_mis_i * \left(\overline{BMI\_ch_i * (1-R_i)} * + \delta_i\right)}{n\_obs_i + n\_mis_i} = BMI\_ch\_ref_i \qquad (1)$$

If we solve the above equation (1) for δ_i, we have that

$$\delta_i = \frac{BMI\_ch\_ref_i * (n\_obs_i + n\_mis_i) - n\_obs_i * (\overline{BMI\_ch_i * R_i})}{n\_mis_i} - \overline{BMI\_ch_i * (1 - R_i)} \qquad (2)$$

The delta values for the average 10-year BMI change are presented in Table S8 below

Table S8: δ-values for the average 10-year BMI change values

Sex	Age 16-24	Age 25-34	Age 35-44	Age 45-54	Age 55-64	Age 65-74
**Men**	-3,3%	-2,5%	-0.3%	0.7%	0,0%	0.6%
**Women**	-4,2%	-4,3%	-2.4%	-1.3%	-0.3%	-1.0%

To calculate the delta values for the 1-year and 5-year BMI change, we worked as follows. For the 1-year BMI change, we estimated the average 1-year BMI change from HSE, by assuming that

$\left(1+\ \mathrm{BMI}_{\mathrm{ch}_{\mathrm{ref}_i}}{}^{10year}(\%)\right)=\left(1+\ \mathrm{BMI\_ch\_ref}_i^{1year}(\%)\right)^{10}$, i.e

$\left(1+\ \mathrm{BMI}_{\mathrm{ch}_{\mathrm{ref}_i}}{}^{1year}(\%)\right)=\sqrt[10]{\left(1+\ \mathrm{BMI\_ch\_ref}_i^{10year}(\%)\right)}$

So, the delta values for the 1-year BMI change are presented in Table S9 below

Table S9: δ-values for the average 1-year BMI change values

Sex	Age 16-24	Age 25-34	Age 35-44	Age 45-54	Age 55-64	Age 65-74
**Men**	-0.8%	-0.6%	-0.1%	0.0%	0.0%	0.2%
**Women**	-1.8%	-0.8%	-0.7%	-0.5%	0.1%	-0.1%

and

$(1+\ \mathrm{BMI\_ch\_ref}_i^{10year}(\%))=\left(1+\ \mathrm{BMI\_ch\_ref}_i^{5year}(\%)\right)^{2}$, i.e

$(1+\ \mathrm{BMI\_ch\_ref}_i^{5year}(\%))=\sqrt{\left(1+\ \mathrm{BMI\_ch\_ref}_i^{10year}(\%)\right)}$

So, the delta values for the 5-year BMI change are presented in Table S10 below

Table S10: δ-values for the average 5-year BMI change values

Sex	Age 16-24	Age 25-34	Age 35-44	Age 45-54	Age 55-64	Age 65-74
**Men**	-2.2%	-1.6%	-0.2%	0.1%	-0.5%	-0.1%
**Women**	-3.4%	-2.6%	-2.1%	-1.8%	-0.9%	-1.7%

3.4: Calculating the odds ratios of the sociodemographic factors for the transition between BMI category within 1-, 5- and 10-year periods

We estimated the odds ratios of the sociodemographic factors for the transition between BMI category within 1-, 5- and 10-year periods, using logistic regression models.

Specifically, to estimate the odds ratios for

a) the transition from normal weight to overweight or obesity, we performed multinomial logistic regression among the normal weight individuals, in which the dependent variable was "0" for those who transitioned to underweight, "1" for those who remained normal weight and "2" for those who transitioned to overweight or obesity. "1" was the reference category and we were interested in the odds ratios in category "2"

b) the transition from overweight or obesity, we performed logistic regression among the overweight individuals, in which the dependent variable was "0" for those who didn't transition and "1" for those who transitioned to obesity

c) the transition from severe to non-severe obesity, we performed logistic regression among individuals with non-severe obesity, in which the dependent variable was "0" for those who didn't transition and "1" for those who transitioned to severe obesity

d) the transition from normal weight to underweight, we performed multinomial logistic regression among the normal weight individuals, in which the dependent variable was "0" for those who transitioned to underweight, "1" for those who remained normal weight and "2" for those who transitioned to overweight or obesity. "1" was the reference category and we were interested in the odds ratios in category "0"

e) remaining in the obesity category, we performed logistic regression among individuals with non-severe obesity, in which the dependent variable was "0" for those who didn't remain and "1" for those who remained in the obesity category.

3.5: Calculation of age-standardised transitions between BMI groups

We calculated the age-standardised transitions between normal weight, overweight and obesity, after taking into consideration the age structure of the English population from ONS between 1998 and 2016. Specifically, we calculated two different weights, which correspond to: i. the proportion of each age group among individuals aged 18-74 (see Table S11) and ii. the prevalence of normal weight, overweight and obesity for each age group (see Table S12)

Table S11: Age structure† of the English population from ONS between 1998 and 2016

Age group (in years)	Proportion among individuals aged 18-74yo
18-24	12.7%
25-34	19.7%
35-44	20.4%
45-54	19.0%
55-64	16.0%
65-74	12.3%

† Average proportion of each age group among individuals aged 18-74, between 1998 and 2016

Table S12: BMI structure† of the English population from HSE between 1998 and 2016, by age group

Age group (in years)	Proportion of normal weight	Proportion of overweight	Proportion of obesity
18-24	60.8%	22.2%	10.7%
25-34	46.4%	33.5%	18.3%
35-44	36.0%	39.0%	24.0%
45-54	30.1%	40.6%	28.8%
55-64	26.5%	42.3%	30.6%
65-74	25.1%	44.0%	30.0%

† Average proportion of normal weight, overweight and obesity for each age group among individuals aged 18-74, between 1998 and 2016

We then combined these weights to calculate the distribution of each age group by BMI group (see Table S13).

Moreover, we estimated the BMI transitions from CALIBER for each age group and then we multiplied each of these transitions (Table S14) with the corresponding proportions from Table S13. In this way, we calculated the standardised transitions by BMI group (see Figure 3 in the paper), after taking into consideration the age and BMI structure of the English population.

Table S13: Age structure[†] of the English population from HSE between 1998 and 2016, by BMI group

Age group (in years)	Proportion of normal weight	Proportion of overweight	Proportion of obesity
18-24	20.8%	7.6%	5.7%
25-34	24.5%	17.7%	15.0%
35-44	19.8%	21.4%	20.5%
45-54	15.3%	20.7%	22.9%
55-64	11.4%	18.1%	20.4%
65-74	8.3%	14.6%	15.4%
Total	100%	100%	100%

[†] Average proportion of each age group among individuals aged 18-74, between 1998 and 2016

Table S14: Estimated transitions from CALIBER from normal weight, overweight and obesity to other BMI groups at one, five and ten years, by age group

		Transitions in the normal weight			Transitions in the overweight			Transitions in the obese		
BMI group	Age group	At 1 year	At 5 years	At 10 years	At 1 year	At 5 years	At 10 years	At 1 year	At 5 years	At 10 years
Underweight	18-24	6.0%	7.7%	7.4%	0.0%	0.4%	0.9%	0.0%	0.0%	0.2%
Normal weight	18-24	84.0%	68.5%	55.2%	18.6%	21.0%	17.5%	0.8%	3.1%	4.1%
Overweight	18-24	9.9%	21.6%	30.4%	68.0%	49.2%	39.2%	16.3%	18.0%	15.8%
Obese	18-24	0.1%	2.2%	7.0%	13.4%	29.4%	42.4%	82.9%	78.8%	79.9%
Underweight	25-34	3.7%	6.1%	6.6%	0.0%	0.2%	0.6%	0.0%	0.0%	0.1%
Normal weight	25-34	84.3%	70.8%	60.5%	17.0%	20.4%	19.0%	0.7%	2.1%	3.3%
Overweight	25-34	11.8%	21.9%	28.8%	71.3%	55.4%	46.0%	15.8%	18.1%	17.0%
Obese	25-34	0.1%	1.3%	4.1%	11.7%	24.0%	34.4%	83.5%	79.8%	79.6%
Underweight	35-44	2.6%	3.8%	4.3%	0.0%	0.1%	0.2%	0.0%	0.0%	0.0%
Normal weight	35-44	83.1%	71.1%	61.8%	14.1%	16.8%	16.2%	0.5%	1.3%	2.0%
Overweight	35-44	14.2%	24.0%	30.6%	73.9%	60.6%	51.6%	14.7%	16.8%	15.7%
Obese	35-44	0.1%	1.1%	3.3%	11.9%	22.5%	31.9%	84.8%	81.8%	82.2%
Underweight	45-54	2.1%	3.3%	4.0%	0.0%	0.1%	0.2%	0.0%	0.0%	0.0%
Normal weight	45-54	82.4%	71.9%	63.8%	12.7%	16.1%	16.5%	0.5%	1.1%	1.8%
Overweight	45-54	15.4%	24.0%	29.8%	76.0%	63.9%	55.6%	14.1%	16.8%	17.1%
Obese	45-54	0.1%	0.9%	2.4%	11.2%	20.0%	27.7%	85.4%	82.1%	81.2%
Underweight	55-64	2.0%	3.4%	4.7%	0.0%	0.1%	0.3%	0.0%	0.0%	0.0%
Normal weight	55-64	82.6%	72.8%	65.3%	11.6%	16.6%	19.1%	0.5%	1.2%	2.3%
Overweight	55-64	15.3%	23.0%	28.0%	78.2%	66.0%	57.3%	14.0%	18.3%	20.2%
Obese	55-64	0.2%	0.8%	2.1%	10.1%	17.3%	23.3%	85.5%	80.5%	77.4%
Underweight	65-74	2.6%	5.5%	8.4%	0.1%	0.3%	0.8%	0.0%	0.0%	0.1%
Normal weight	65-74	83.6%	74.6%	68.0%	12.3%	20.8%	26.8%	0.5%	2.0%	5.3%
Overweight	65-74	13.6%	19.2%	22.2%	79.0%	64.8%	54.9%	15.3%	23.0%	27.0%
Obese	65-74	0.2%	0.6%	1.4%	8.7%	14.1%	17.4%	84.2%	74.9%	67.6%

3.6. Converting an odds ratio to a relative risk (for the online calculator)

In Figure 5, we calculated the absolute risk of transitioning to a higher BMI category over 10 years, by initial BMI category, age, sex and social deprivation non-parametrically. We wanted to do the same, this time accounting for ethnicity as well in our online calculator. If we did that non-parametrically, we would have near positivity issues; this means that in some strata defined by all potential categories of the sociodemographic factors, the number of individuals would be very small. For this reason, we used a semi-parametric approach: We calculated the (non-parametric) 10-year BMI transition by age, sex, index of multiple deprivation and current BMI overall is calculated in white individuals and then multiplied by the relative risk of transitions of ethnicity from Figure 4. More specifically, we converted the corresponding odds ratios from Figure 4 to relative risks using the formula from [1], see below

$$\text{Relative risk} = \frac{\text{odds ratio}}{1 - \mathrm{p}_0 + (\mathrm{p}_0 \times \text{odds ratio})}$$

where p_0 is the baseline risk, i.e. the risk in each cell of Figure S5 which is the absolute risk of transitioning to a higher BMI category over 10 years, by initial BMI category, age, sex and social deprivation non-parametrically in white individuals only.

References

[1] Grant RL. Converting an odds ratio to a range of plausible relative risks for better communication of research findings. BMJ 2014; 348:f7450.

Section 4: Additional Results

Figure S5: Absolute risk for the transitions from normal weight to overweight or obesity, from overweight to obesity and from non-severe to severe obesity in 10 years in white individuals only, by age, social deprivation, sex and initial BMI. N=1,048,878 individuals: across the 900 strata - there are at least 1000 participants in 48% (432) of strata, and >100 participants in 99% (889) of the strata

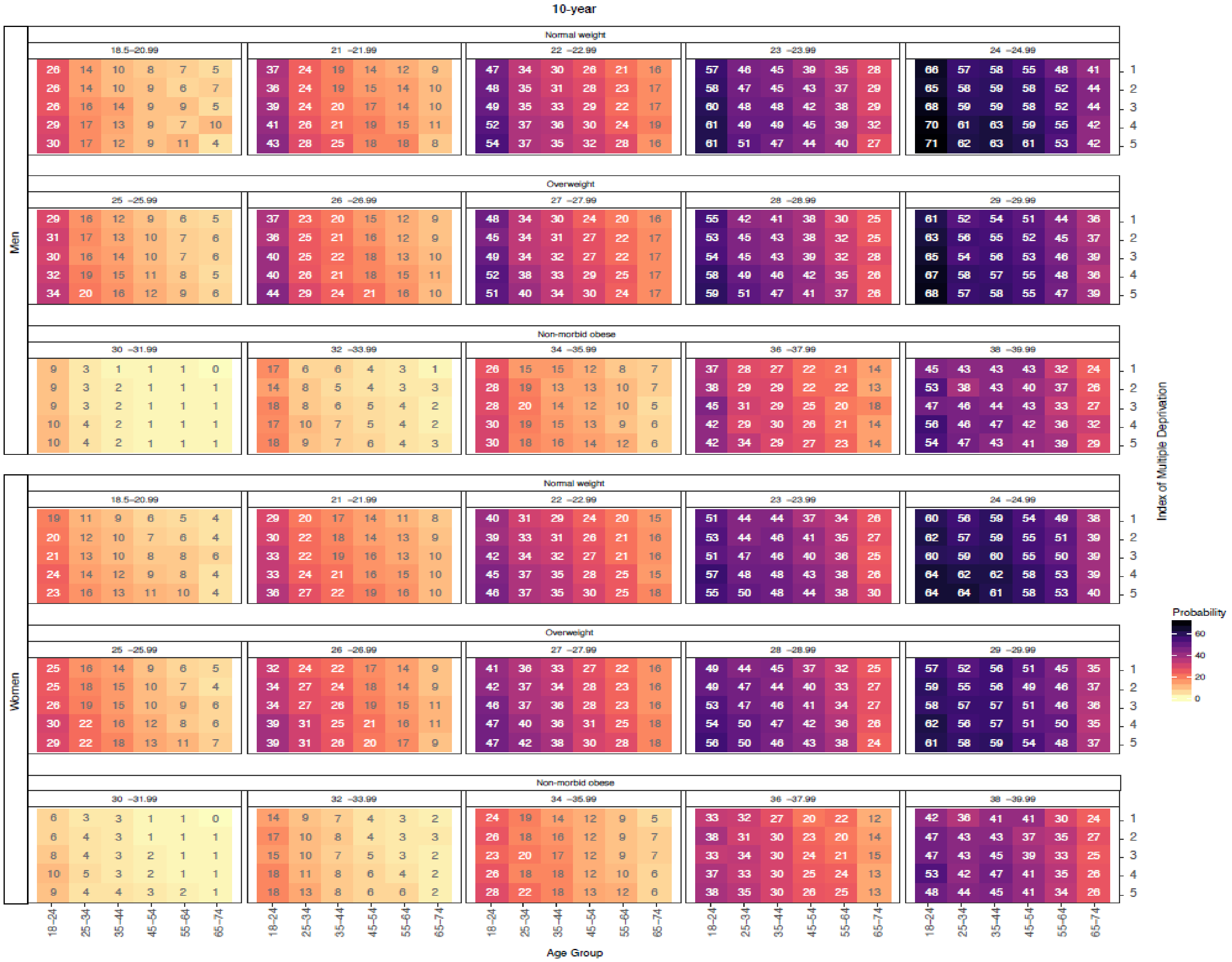

Figure S6: Weight trajectories between 1998 to 2016 in CALIBER. Average* of initial weight (beginning of each line; measured between 1998 and 2008) and estimated average of weight after 10 years (end of each line), by sex and age (youngest cohort 18-24 years old; oldest cohort: 65-74 years old)

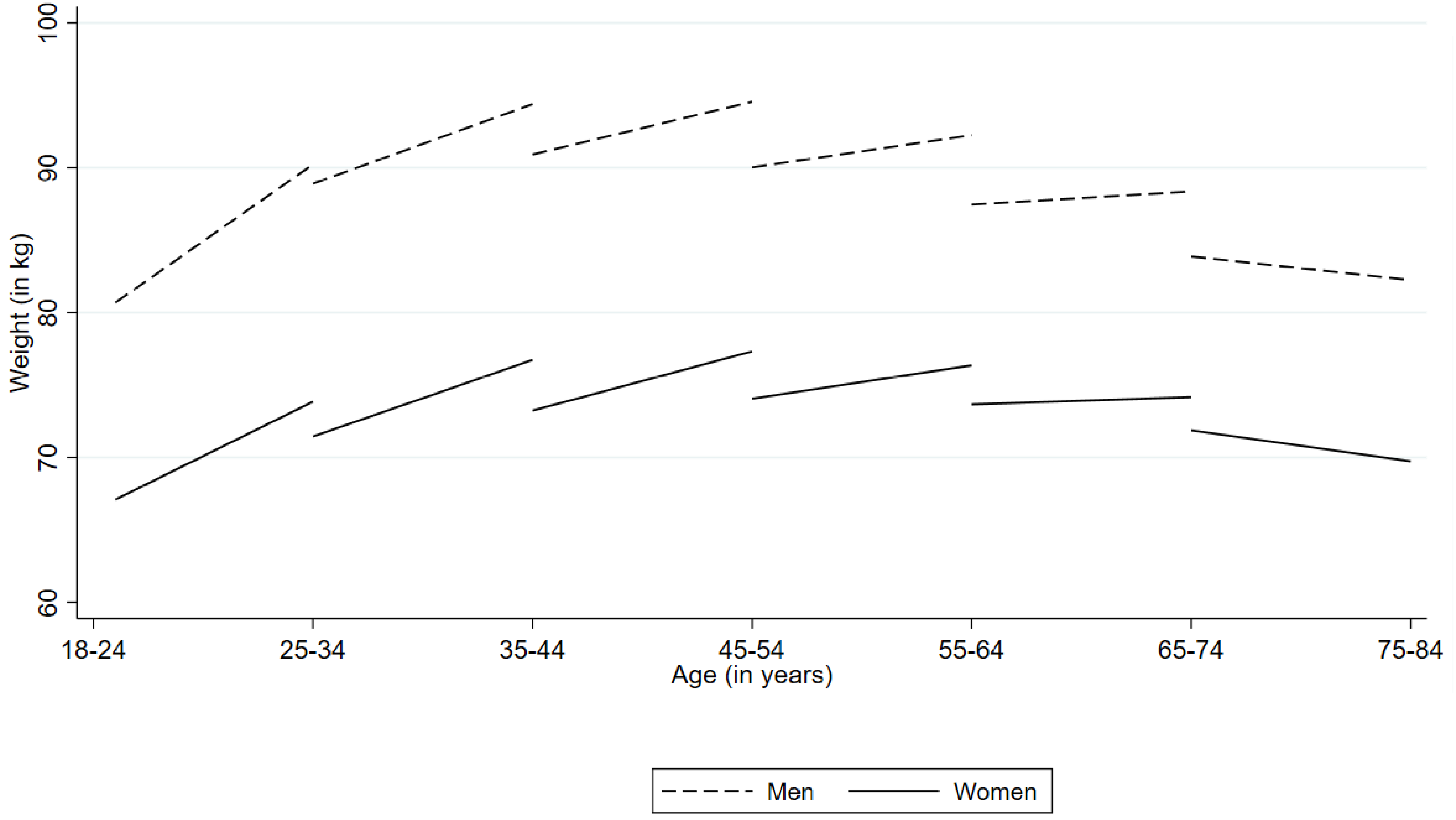

*Estimated averages of weight have been calculated from the complete-case analysis of 10-year weight change

Figure S7: Distribution of the 1-, 5- and 10-year weight change* between 1998 and 2016 in England, by age group, separately in men (upper panel: with average height of 1.76m) and in women (lower panel: with height of 1.62m)

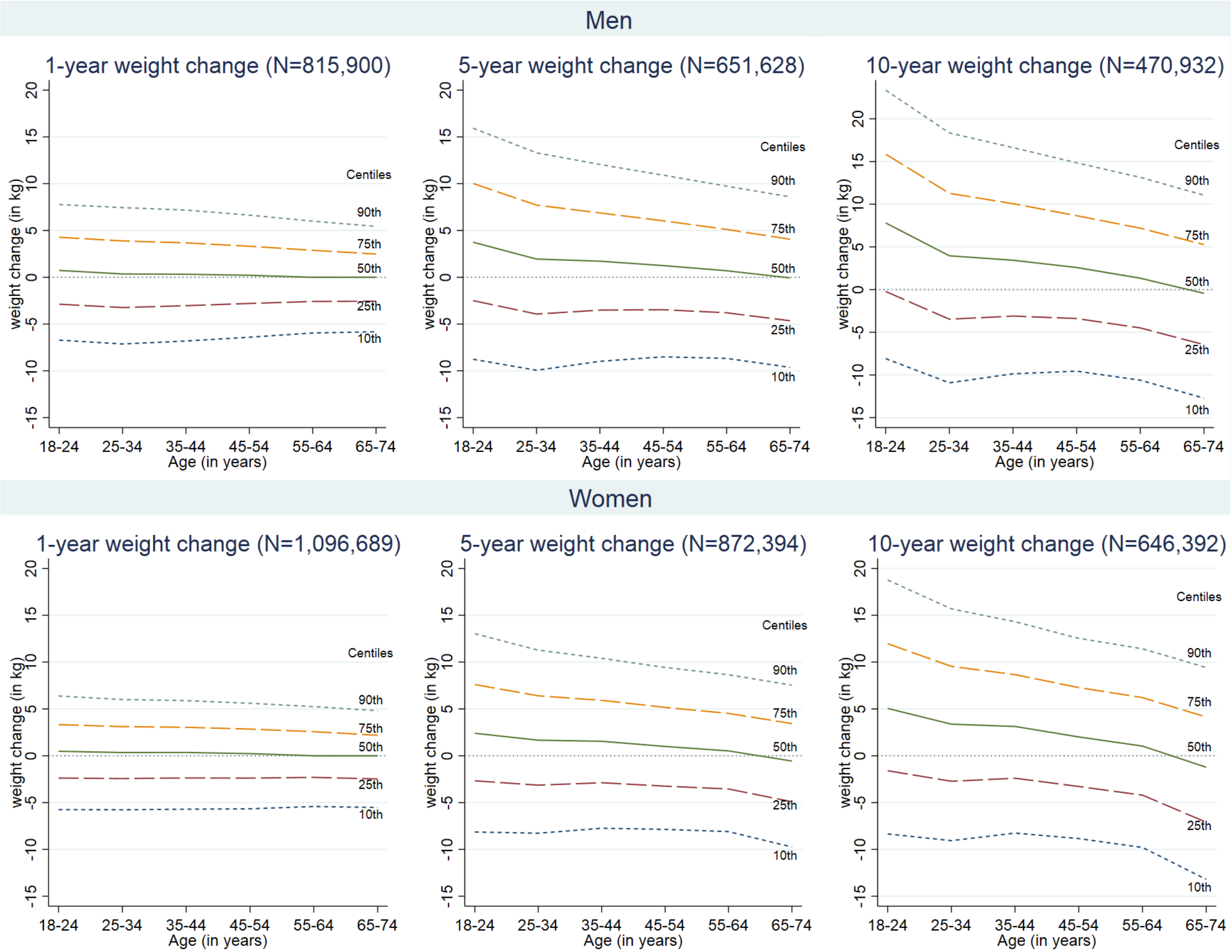

*Weight change is calculated indirectly from sex specific BMI change for men with height of 1.76m (median in CALIBER) and women with height of 1.62m (median in CALIBER)

Figure S8: Absolute risks and odds ratios* of transitioning at ten years from normal weight to overweight or obesity, from overweight to obesity and from non-severe to severe obesity, by age, sex, ethnicity, social deprivation and region among young adults aged 18-24 years old

	Normal weight to overweight or obesity				Overweight to obesity				Non severe obesity to severe obesity			
	BMI at baseline¶	N of transition‡	Absolute Risk(%)	Odds Ratio	BMI at baseline¶	N of transition‡	Absolute Risk(%)	Odds Ratio	BMI at baseline¶	N of transition‡	Absolute Risk(%)	Odds Ratio
**Sex**												
women	21.7	11986	34	1.00 - Reference	27.1	5322	41	1.00 - Reference	33.6	1807	21	1.00 - Reference
men	21.8	7258	43	1.35 (1.18, 1.53)	27	2948	45	0.95 (0.93, 0.97)	33.6	900	24	0.99 (0.95, 1.02)
**Ethnicity**												
White	21.7	17734	37	1.00 - Reference	27.1	7656	42	1.00 - Reference	33.6	2515	22	1.00 - Reference
Mixed/Other	21.5	746	38	1.05 (0.85, 1.30)	27	276	43	0.89 (0.84, 0.95)	33.5	87	22	0.86 (0.77, 0.97)
Asian	21.6	346	37	0.98 (0.74, 1.30)	27	139	44	1.01 (0.90, 1.13)	33.4	45	22	0.90 (0.73, 1.10)
Black	21.7	418	49	1.40 (1.00, 1.97)	26.9	199	56	1.13 (1.05, 1.23)	33.5	60	28	0.96 (0.83, 1.10)
**IMD (from less to more deprived)**												
1st quintile	21.7	3499	35	1.00 - Reference	27	1294	39	1.00 - Reference	33.4	326	20	1.00 - Reference
2nd quintile	21.7	3627	35	1.03 (0.93, 1.15)	27	1411	39	1.06 (1.03, 1.10)	33.6	462	22	1.10 (1.04, 1.16)
3rd quintile	21.7	3836	36	1.08 (0.94, 1.25)	27	1642	42	1.13 (1.09, 1.16)	33.6	505	21	1.16 (1.09, 1.22)
4th quintile	21.7	4278	39	1.19 (1.04, 1.37)	27.1	1989	45	1.24 (1.20, 1.27)	33.7	659	23	1.26 (1.19, 1.34)
5th quintile	21.7	4004	40	1.24 (1.10, 1.39)	27.1	1934	44	1.32 (1.28, 1.36)	33.8	755	23	1.34 (1.27, 1.43)
**Region**												
London	21.6	2534	36	1.00 - Reference	27	1010	41	1.00 - Reference	33.6	308	22	1.00 - Reference
South West	21.7	1863	36	1.07 (0.96, 1.20)	27.1	865	42	1.04 (1.00, 1.08)	33.7	301	20	1.05 (0.98, 1.12)
South Central	21.7	2442	37	1.06 (0.92, 1.23)	27	1024	41	1.01 (0.97, 1.06)	33.7	365	23	1.03 (0.96, 1.10)
South East	21.7	2250	36	1.08 (0.96, 1.23)	27.1	940	42	1.06 (1.02, 1.10)	33.6	319	23	1.07 (0.99, 1.15)
East	21.7	2031	36	0.99 (0.90, 1.10)	27	837	40	0.99 (0.95, 1.03)	33.6	259	20	0.98 (0.91, 1.06)
West Midlands	21.7	2518	37	1.04 (0.88, 1.22)	27.1	1093	43	1.04 (1.00, 1.08)	33.7	328	21	1.06 (0.99, 1.13)
C/N East†	21.8	3642	39	1.12 (0.98, 1.27)	27	1569	44	1.03 (0.99, 1.07)	33.6	542	24	1.05 (0.98, 1.13)
North West	21.8	1964	38	1.13 (0.98, 1.31)	27.1	932	43	1.15 (1.10, 1.21)	33.5	285	22	1.16 (1.07, 1.25)

Odds ratio axis (each panel): .5 1 2 4

* Mutually adjusted for BMI (at baseline), sex, Index of multiple deprivation (IMD – quintiles; in categories), ethnicity, region, use of diuretics, prevalence of CVD, cancer, diabetes, hypertension, mental health disorders (dep depression, anxiety, stress, phobia, schizophrenia, bipolar disorder, affective disorder) and other chronic diseases [HIV, chronic obstructive pulmonary disease, neurological (dementia), rheumatological (rheumatoid arthritis, gout, systemic lupus erythematosus), gastro-intestinal (inflammatory bowel disease) and renal (chronic kidney disease, renal failure)]
†Central/North East
‡N of individuals who transitioned to higher BMI categories
¶Mean BMI levels at baseline

Figure S9: Absolute risks and odds ratios* of (i) transitioning from normal weight to underweiht and (ii) remaining in obesity at ten years, by age, sex, ethnicity, social deprivation and region

Normal weight to underweight

N: 400 367

	BMI at baseline¶	N of transition‡	Absolute Risk(%)	Odds Ratio
**Age (in years)**				
18 - 24	21.7	3840	7	0.57 (0.53, 0.62)
25 - 34	22.1	4299	6	0.58 (0.52, 0.65)
35 - 44	22.4	3684	4	0.42 (0.38, 0.46)
45 - 54	22.6	3163	4	0.42 (0.39, 0.46)
55 - 64	22.8	3211	5	0.53 (0.50, 0.57)
65 - 74	22.8	4144	8	1.00 - Reference
**Sex**				
women	22.3	16446	6	1.00 - Reference
men	22.8	5895	4	0.88 (0.84, 0.92)
**Ethnicity**				
White	22.4	20823	6	1.00 - Reference
Mixed/Other	22.3	968	6	1.07 (0.99, 1.16)
Asian	22.3	312	6	1.08 (0.88, 1.33)
Black	22.5	238	4	0.88 (0.71, 1.09)
**IMD (from less to more deprived)**				
1st quintile	22.4	5821	6	1.00 - Reference
2nd quintile	22.5	4952	5	0.97 (0.92, 1.04)
3rd quintile	22.4	4576	6	0.99 (0.92, 1.06)
4th quintile	22.4	3713	5	0.96 (0.89, 1.04)
5th quintile	22.3	3279	6	1.02 (0.94, 1.11)
**Region**				
London	22.4	3239	6	1.00 - Reference
South West	22.4	2340	6	0.91 (0.84, 0.98)
South Central	22.4	3029	6	0.96 (0.89, 1.03)
South East	22.4	2818	6	0.93 (0.85, 1.02)
East	22.4	2645	6	0.96 (0.89, 1.04)
West Midlands	22.5	2664	5	0.93 (0.85, 1.01)
C/N East†	22.5	3611	6	0.95 (0.89, 1.03)
North West	22.4	1995	5	0.88 (0.81, 0.97)

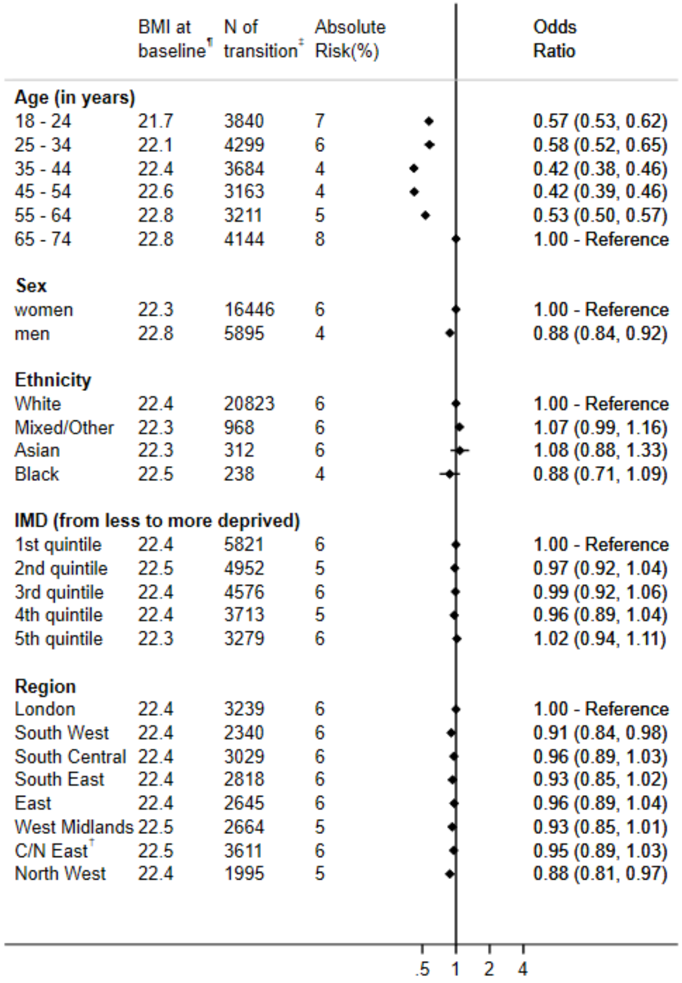

Remaining in obesity category

N: 297 642

	BMI at baseline¶	N of remaining‡‡	Absolute Risk(%)	Odds Ratio
**Age (in years)**				
18 - 24	35.1	11722	82	1.76 (1.58, 1.95)
25 - 34	35.1	27091	82	1.72 (1.57, 1.88)
35 - 44	35	53948	84	2.09 (1.97, 2.22)
45 - 54	34.7	62395	83	1.97 (1.87, 2.08)
55 - 64	34.3	54007	79	1.62 (1.55, 1.69)
65 - 74	33.8	30192	70	1.00 - Reference
**Sex**				
women	35.2	137220	81	1.00 - Reference
men	33.9	102135	80	1.17 (1.14, 1.21)
**Ethnicity**				
White	34.6	226631	80	1.00 - Reference
Mixed/Other	33.8	5295	76	0.88 (0.80, 0.96)
Asian	34.4	2321	80	0.89 (0.77, 1.01)
Black	34.8	5108	83	1.05 (0.94, 1.17)
**IMD (from less to more deprived)**				
1st quintile	34.1	44600	78	1.00 - Reference
2nd quintile	34.4	49260	80	1.04 (1.00, 1.08)
3rd quintile	34.6	49561	80	1.05 (1.01, 1.09)
4th quintile	34.8	50011	82	1.09 (1.04, 1.14)
5th quintile	35.2	45923	83	1.09 (1.04, 1.13)
**Region**				
London	34.6	27981	80	1.00 - Reference
South West	34.7	28892	81	1.09 (1.03, 1.15)
South Central	34.6	31659	80	1.00 (0.95, 1.05)
South East	34.7	30090	81	1.06 (1.00, 1.12)
East	34.5	25414	79	0.98 (0.93, 1.04)
West Midlands	34.6	30156	81	1.05 (1.00, 1.11)
C/N East†	34.6	40291	80	1.02 (0.98, 1.06)
North West	34.7	24872	81	1.12 (1.06, 1.18)

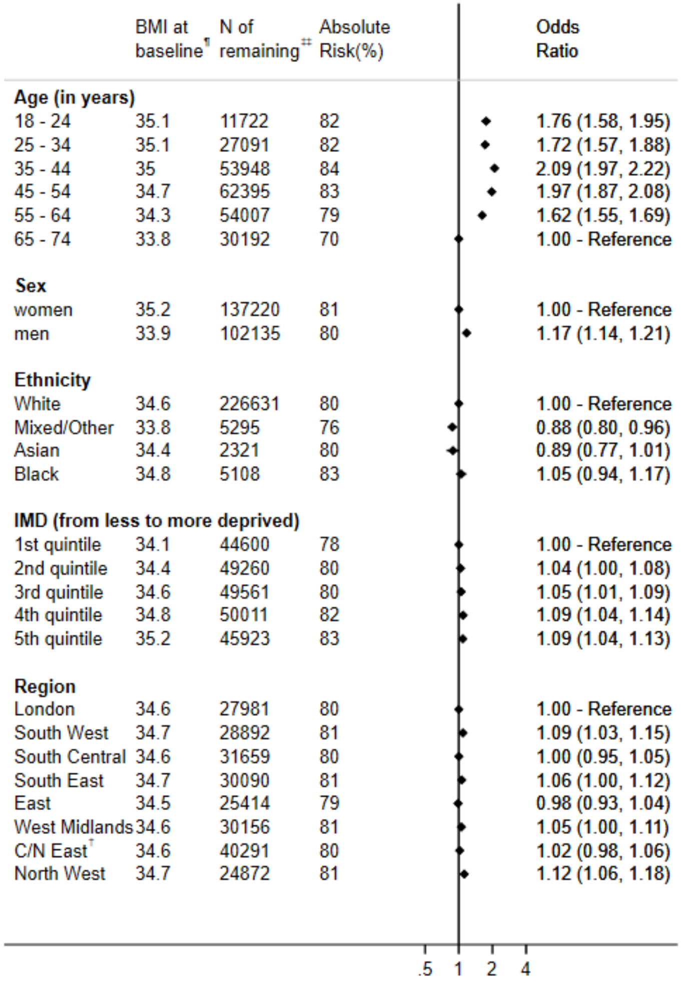

* Mutually adjusted for BMI (at baseline), age group, sex, Index of multiple deprivation (IMD – quintiles; in categories), ethnicity, region, use of diuretics, prevalence of CVD, cancer, diabetes, hypertension, mental health disorders (dep depression, anxiety, stress, phobia, schizophrenia, bipolar disorder, affective disorder) and other chronic diseases [HIV, chronic obstructive pulmonary disease, neurological (dementia), rheumatological (rheumatoid arthritis, gout, systemic lupus erythematosus), gastro-intestinal (inflammatory bowel disease) and renal (chronic kidney disease, renal failure)]

†Central/North East

‡ N of individuals who transitioned to underweight

‡‡ N of individuals who remained in obesity category

¶Mean BMI levels at baseline

Figure S10: Absolute risks and odds ratios* of transitioning at one year from normal weight to overweight or obesity, from overweight to obesity and from non-severe to severe obesity, by age, sex, ethnicity, social deprivation and region

Normal weight to overweight or obesity

	BMI at baseline¶	N of transition‡	Absolute Risk(%)	Odds ratio
**Age (in years)**				
18 - 24	21.7	10577	10	1.58 (1.51, 1.65)
25 - 34	22.1	16124	12	1.43 (1.36, 1.51)
35 - 44	22.4	18841	14	1.42 (1.34, 1.50)
45 - 54	22.6	18845	15	1.37 (1.30, 1.44)
55 - 64	22.7	15883	15	1.21 (1.16, 1.26)
65 - 74	22.7	12752	14	1.00 - Reference
**Sex**				
women	22.2	53722	12	1.00 - Reference
men	22.7	39300	17	1.11 (1.09, 1.13)
**Ethnicity**				
White	22.4	85469	14	1.00 - Reference
Mixed/Other	22.3	4062	13	0.93 (0.89, 0.98)
Asian	22.2	1584	13	0.97 (0.89, 1.05)
Black	22.5	1907	15	1.02 (0.92, 1.13)
**IMD (from less to more deprived)**				
1st quintile	22.4	22176	13	1.00 - Reference
2nd quintile	22.4	20348	13	1.04 (1.01, 1.07)
3rd quintile	22.4	18793	14	1.06 (1.03, 1.09)
4th quintile	22.3	17244	14	1.11 (1.07, 1.14)
5th quintile	22.3	14461	14	1.13 (1.09, 1.17)
**Region**				
London	22.3	13531	13	1.00 - Reference
South West	22.4	10593	14	1.06 (1.02, 1.10)
South Central	22.4	11957	13	1.01 (0.97, 1.06)
South East	22.4	11721	13	1.05 (1.01, 1.09)
East	22.3	10825	13	0.98 (0.95, 1.02)
West Midlands	22.4	11106	14	1.01 (0.97, 1.05)
C/N East†	22.4	13881	14	1.02 (0.98, 1.06)
North West	22.4	9408	14	1.06 (1.02, 1.11)

.5 1 2 4

Overweight to obesity

	BMI at baseline¶	N of transition‡	Absolute Risk(%)	Odds ratio
**Age (in years)**				
18 - 24	27.1	5194	13	2.15 (2.05, 2.26)
25 - 34	27.1	9217	11	1.66 (1.59, 1.74)
35 - 44	27.2	14338	12	1.58 (1.52, 1.64)
45 - 54	27.3	16489	11	1.39 (1.34, 1.44)
55 - 64	27.3	14457	10	1.18 (1.14, 1.23)
65 - 74	27.3	10787	9	1.00 - Reference
**Sex**				
women	27.2	34672	11	1.00 - Reference
men	27.3	35810	11	0.95 (0.93, 0.97)
**Ethnicity**				
White	27.3	64915	11	1.00 - Reference
Mixed/Other	27.2	2562	9	0.87 (0.82, 0.93)
Asian	27.2	1052	11	0.98 (0.89, 1.09)
Black	27.4	1953	12	0.99 (0.92, 1.07)
**IMD (from less to more deprived)**				
1st quintile	27.2	14594	10	1.00 - Reference
2nd quintile	27.3	15105	10	1.05 (1.01, 1.09)
3rd quintile	27.3	14750	11	1.08 (1.04, 1.13)
4th quintile	27.3	13960	11	1.12 (1.09, 1.15)
5th quintile	27.3	12073	12	1.14 (1.10, 1.18)
**Region**				
London	27.2	9793	11	1.00 - Reference
South West	27.3	8464	11	1.03 (0.99, 1.08)
South Central	27.3	8776	10	1.01 (0.96, 1.05)
South East	27.3	8948	11	1.03 (0.99, 1.08)
East	27.2	7890	10	0.99 (0.94, 1.03)
West Midlands	27.3	8522	11	1.00 (0.96, 1.04)
C/N East†	27.3	10870	11	0.99 (0.95, 1.03)
North West	27.3	7219	11	1.03 (0.98, 1.08)

.5 1 2 4

Non severe obesity to severe obesity

	BMI at baseline¶	N of transition‡	Absolute Risk(%)	Odds ratio
**Age (in years)**				
18 - 24	33.6	1202	5	2.39 (2.14, 2.67)
25 - 34	33.6	2138	4	1.89 (1.73, 2.07)
35 - 44	33.5	3040	3	1.70 (1.55, 1.85)
45 - 54	33.4	3162	3	1.45 (1.32, 1.58)
55 - 64	33.3	2456	2	1.24 (1.15, 1.34)
65 - 74	33.2	1469	2	1.00 - Reference
**Sex**				
women	33.6	8701	4	1.00 - Reference
men	33.1	4766	2	0.95 (0.91, 1.00)
**Ethnicity**				
White	33.4	12586	3	1.00 - Reference
Mixed/Other	33	278	2	0.82 (0.71, 0.96)
Asian	33.3	160	3	0.89 (0.73, 1.08)
Black	33.5	443	3	0.95 (0.82, 1.09)
**IMD (from less to more deprived)**				
1st quintile	33.2	2024	2	1.00 - Reference
2nd quintile	33.3	2507	3	1.06 (0.98, 1.15)
3rd quintile	33.4	2808	3	1.07 (0.99, 1.15)
4th quintile	33.5	2958	3	1.07 (1.00, 1.16)
5th quintile	33.6	3170	4	1.11 (1.03, 1.20)
**Region**				
London	33.4	1719	3	1.00 - Reference
South West	33.5	1777	3	1.06 (0.96, 1.17)
South Central	33.4	1693	3	1.07 (0.98, 1.17)
South East	33.4	1668	3	1.01 (0.92, 1.11)
East	33.4	1418	3	0.98 (0.90, 1.08)
West Midlands	33.4	1592	3	1.04 (0.95, 1.14)
C/N East†	33.4	2215	3	1.06 (0.98, 1.15)
North West	33.4	1385	3	1.05 (0.95, 1.15)

.5 1 2 4

* Mutually adjusted for BMI (at baseline), age group, sex, Index of multiple deprivation (IMD – quintiles; in categories), ethnicity, region, use of diuretics, prevalence of CVD, cancer, diabetes, hypertension, mental health disorders (dep depression, anxiety, stress, phobia, schizophrenia, bipolar disorder, affective disorder) and other chronic diseases [HIV, chronic obstructive pulmonary disease, neurological (dementia), rheumatological (rheumatoid arthritis, gout, systemic lupus erythematosus), gastro-intestinal (inflammatory bowel disease) and renal (chronic kidney disease, renal failure)]

†Central/North East

‡N of individuals who transitioned to higher BMI categories

¶Mean BMI levels at baseline

Figure S11: Absolute risks and odds ratios* of transitioning at five years from normal weight to overweight or obesity, from overweight to obesity and from non-severe to severe obesity, by age, sex, ethnicity, social deprivation and region

Normal weight to overweight or obesity

	BMI at baseline¶	N of transition‡	Absolute Risk(%)	Odds ratio
**Age (in years)**				
18 - 24	21.7	18854	24	2.88 (2.73, 3.03)
25 - 34	22.1	22610	23	2.07 (1.98, 2.16)
35 - 44	22.4	27742	25	1.89 (1.82, 1.97)
45 - 54	22.6	25229	25	1.61 (1.55, 1.67)
55 - 64	22.7	20830	24	1.35 (1.29, 1.40)
65 - 74	22.8	14119	20	1.00 - Reference
**Sex**				
women	22.2	77827	22	1.00 - Reference
men	22.7	51557	28	1.12 (1.09, 1.15)
**Ethnicity**				
White	22.4	119902	24	1.00 - Reference
Mixed/Other	22.3	5104	22	0.92 (0.87, 0.97)
Asian	22.2	1937	23	0.94 (0.83, 1.07)
Black	22.5	2441	27	1.06 (0.98, 1.15)
**IMD (from less to more deprived)**				
1st quintile	22.4	30498	22	1.00 - Reference
2nd quintile	22.4	28631	23	1.05 (1.02, 1.08)
3rd quintile	22.4	26039	24	1.08 (1.06, 1.11)
4th quintile	22.4	24066	25	1.16 (1.13, 1.19)
5th quintile	22.3	20150	25	1.21 (1.16, 1.26)
**Region**				
London	22.3	17380	23	1.00 - Reference
South West	22.4	14321	24	1.08 (1.05, 1.12)
South Central	22.4	16805	23	1.04 (0.99, 1.08)
South East	22.4	15973	23	1.07 (1.03, 1.12)
East	22.4	14991	23	1.02 (0.98, 1.06)
West Midlands	22.4	15855	24	1.05 (1.01, 1.10)
C/N East†	22.4	20375	25	1.04 (1.00, 1.08)
North West	22.4	13684	26	1.12 (1.08, 1.16)

Overweight to obesity

	BMI at baseline¶	N of transition‡	Absolute Risk(%)	Odds ratio
**Age (in years)**				
	27.1	8741	29	3.48 (3.31, 3.66)
	27.1	14484	24	2.40 (2.31, 2.49)
	27.2	23069	23	2.06 (1.98, 2.14)
	27.3	24347	20	1.64 (1.59, 1.70)
	27.3	20753	17	1.31 (1.27, 1.35)
	27.3	14070	14	1.00 - Reference
**Sex**				
	27.2	52983	20	1.00 - Reference
	27.3	52481	19	0.94 (0.92, 0.96)
**Ethnicity**				
	27.3	98279	20	1.00 - Reference
	27.1	3398	17	0.83 (0.77, 0.88)
	27.2	1396	20	0.95 (0.86, 1.04)
	27.3	2391	21	0.93 (0.87, 1.00)
**IMD (from less to more deprived)**				
	27.2	22314	18	1.00 - Reference
	27.3	22650	19	1.04 (1.01, 1.07)
	27.3	21748	20	1.08 (1.05, 1.11)
	27.3	20759	21	1.14 (1.10, 1.18)
	27.3	17993	23	1.19 (1.16, 1.23)
**Region**				
	27.2	13255	19	1.00 - Reference
	27.3	12527	20	1.08 (1.03, 1.12)
	27.3	13236	19	1.02 (0.98, 1.06)
	27.3	12811	19	1.05 (1.00, 1.10)
	27.2	11856	19	1.02 (0.98, 1.06)
	27.3	13038	20	1.04 (1.00, 1.08)
	27.3	17035	20	1.02 (0.97, 1.06)
	27.3	11706	21	1.13 (1.09, 1.18)

Non severe obesity to severe obesity

	BMI at baseline¶	N of transition‡	Absolute Risk(%)	Odds ratio
**Age (in years)**				
	33.6	2487	13	4.32 (3.91, 4.77)
	33.6	3733	9	2.69 (2.50, 2.89)
	33.5	5526	8	2.18 (2.03, 2.34)
	33.4	5612	6	1.77 (1.65, 1.89)
	33.3	4112	5	1.36 (1.27, 1.45)
	33.1	2115	3	1.00 - Reference
**Sex**				
	33.6	14995	8	1.00 - Reference
	33.1	8590	5	0.93 (0.89, 0.97)
**Ethnicity**				
	33.4	22223	7	1.00 - Reference
	33	452	5	0.75 (0.66, 0.85)
	33.2	239	6	0.83 (0.69, 0.99)
	33.5	671	7	0.87 (0.76, 1.00)
**IMD (from less to more deprived)**				
	33.2	3672	5	1.00 - Reference
	33.3	4264	6	1.03 (0.97, 1.10)
	33.4	4787	6	1.11 (1.05, 1.17)
	33.5	5443	7	1.14 (1.08, 1.21)
	33.6	5419	8	1.21 (1.14, 1.28)
**Region**				
	33.4	2876	6	1.00 - Reference
	33.5	2995	7	1.04 (0.96, 1.13)
	33.4	2962	6	1.02 (0.95, 1.09)
	33.4	2938	6	1.02 (0.95, 1.10)
	33.3	2338	6	0.97 (0.90, 1.05)
	33.3	2914	7	1.04 (0.97, 1.12)
	33.4	3884	7	1.01 (0.94, 1.09)
	33.4	2678	7	1.11 (1.03, 1.20)

Odds ratio axis: .5 1 2 4

* Mutually adjusted for BMI (at baseline), age group, sex, Index of multiple deprivation (IMD – quintiles; in categories), ethnicity, region, use of diuretics, prevalence of CVD, cancer, diabetes, hypertension, mental health disorders (dep depression, anxiety, stress, phobia, schizophrenia, bipolar disorder, affective disorder) and other chronic diseases [HIV, chronic obstructive pulmonary disease, neurological (dementia), rheumatological (rheumatoid arthritis, gout, systemic lupus erythematosus), gastro-intestinal (inflammatory bowel disease) and renal (chronic kidney disease, renal failure)]

†Central/North East

‡N of individuals who transitioned to higher BMI categories

¶Mean BMI levels at baseline

Figure S12: Absolute risk for the transitions from normal weight to overweight or obesity, from overweight to obesity and from non-severe to severe obesity in 1 year, by age, social deprivation, sex and initial BMI. N=1,912,589 individuals: across the 900 strata - there are at least 1000 participants in 77% (689) of strata, and >100 participants in 99.7% (897) of the strata

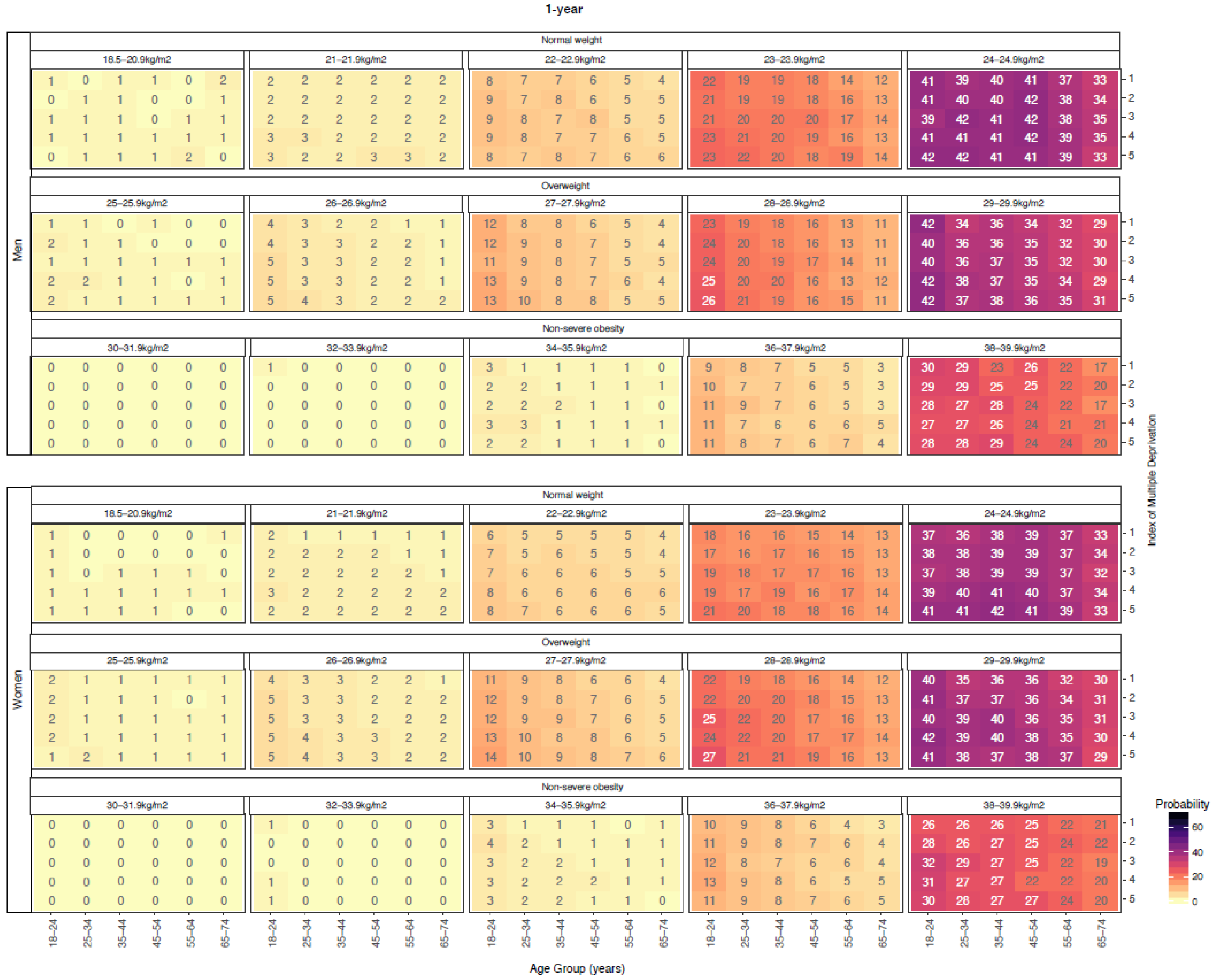

Figure S13: Absolute risk for the transitions from normal weight to overweight or obesity, from overweight to obesity and from non-severe to severe obesity in 5 years, by age, social deprivation, sex and initial BMI. N=1,524,022 individuals: across the 900 strata - there are at least 1000 participants in 77% (589) of strata, and >100 participants in 99.6% (896) of the strata

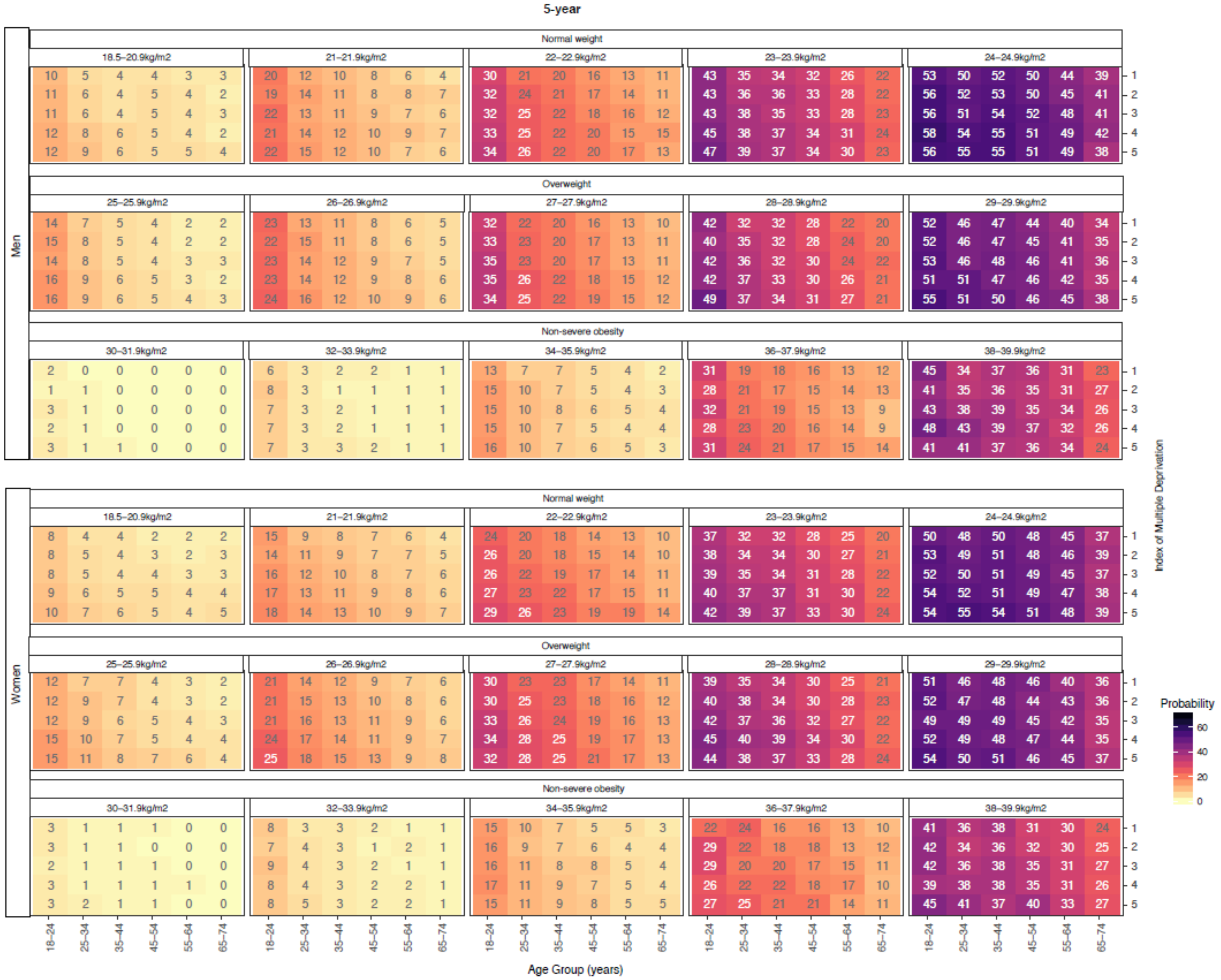

Sensitivity analyses

We first applied sensitivity analysis and created two extra datasets for the calculation of the 10-year BMI change using the multiple imputation with delta adjustment, using a different seed and we compared the 25th, 50th and 75th centile by age and sex with the BMI change from the main analysis (Table S15).

Moreover, regarding the models, we applied the same analysis, i. without controlling for the prevalent chronic conditions (Figure S14), ii. excluding individuals with prevalent chronic diseases at baseline (Figure S15) and iii. excluding individuals with prevalent chronic diseases and those who developed chronic conditions during follow-up (Figure S16). iv. excluding all missing values (complete case analysis) (Figure S17), v. without accounting for the delta values after the multiple imputation (Figure S18), vi. Including smoking status as an extra covariate in the models (Table S16) as well as presenting all the results separately for never, former and current smokers.(Table S17)

Table S15: Comparison of the 25th, 50th and 75th centile of the 10-year BMI change (%) from the main analysis with 2 extra datasets after multiple imputaion (with different seed) with delta adjustemnt, separately in men and women

Men

	10-year BMI change used in this analysis			10-year BMI change (sensitivity 1)			10-year BMI change (sensitivity 2)		
	25th centile	50th centile	75th centile	25th centile	50th centile	75th centile	25th centile	50th centile	75th centile
**18-24 years**	-0.30%	10.76%	21.96%	-0.29%	10.74%	21.92%	-0.40%	10.73%	22.00%
**25-34 years**	-4.20%	4.91%	14.13%	-4.20%	4.92%	14.14%	-4.20%	4.94%	14.14%
**35-44 years**	-3.62%	4.10%	11.93%	-3.60%	4.09%	11.93%	-3.61%	4.09%	11.93%
**45-54 years**	-3.90%	3.05%	10.18%	-3.91%	3.04%	10.19%	-3.90%	3.10%	10.19%
**55-64 years**	-5.21%	1.57%	8.53%	-5.19%	1.57%	8.51%	-5.19%	1.57%	8.52%
**65-74 years**	-7.61%	-0.23%	6.35%	-7.60%	-0.52%	6.35%	-7.61%	-0.18%	6.35%

Women

	10-year BMI change used in this analysis			10-year BMI change (sensitivity 1)			10-year BMI change (sensitivity 2)		
	25th centile	50th centile	75th centile	25th centile	50th centile	75th centile	25th centile	50th centile	75th centile
**18-24 years**	-2.52%	8.25%	19.45%	-2.50%	8.25%	19.45%	-2.51%	8.26%	19.49%
**25-34 years**	-4.20%	5.21%	14.63%	-4.06%	5.24%	14.63%	-4.06%	5.22%	14.62%
**35-44 years**	-3.48%	4.64%	12.87%	-3.47%	4.65%	12.87%	-3.49%	4.09%	12.87%
**45-54 years**	-4.61%	2.92%	10.54%	-4.60%	2.92%	10.54%	-4.59%	2.91%	10.54%
**55-64 years**	-5.86%	1.48%	8.88%	-5.86%	1.49%	8.87%	-5.86%	1.48%	8.87%
**65-74 years**	-9.86%	-1.74%	5.94%	-9.85%	-1.75%	5.94%	-9.86%	-1.74%	5.94%

Figure S14: Absolute risks and odds ratios* of transitioning at ten years from normal weight to overweight or obesity, from overweight to obesity and from non-severe to severe obesity, by age, sex, ethnicity, social deprivation and region (without adjusting for chronic diseases)

Normal weight to overweight or obesity
N: 400 367

	BMI at baseline¶	N of transition‡	Absolute Risk(%)	Odds Ratio
**Age (in years)**				
18 - 24	21.7	19244	37	3.95 (3.58, 4.35)
25 - 34	22.1	22565	34	2.42 (2.29, 2.56)
35 - 44	22.4	29276	34	2.16 (2.07, 2.25)
45 - 54	22.6	25369	32	1.75 (1.69, 1.82)
55 - 64	22.8	20143	30	1.44 (1.38, 1.50)
65 - 74	22.8	11527	24	1.00 - Reference
**Sex**				
women	22.3	79426	30	1.00 - Reference
men	22.8	48698	36	1.10 (1.06, 1.14)
**Ethnicity**				
White	22.4	119802	32	1.00 - Reference
Mixed/Other	22.3	4672	31	0.90 (0.85, 0.96)
Asian	22.3	1659	31	0.92 (0.84, 1.00)
Black	22.5	1991	37	1.12 (1.03, 1.23)
**IMD (from less to more deprived)**				
1st quintile	22.4	30808	30	1.00 - Reference
2nd quintile	22.5	28546	31	1.07 (1.04, 1.10)
3rd quintile	22.4	25562	32	1.10 (1.06, 1.14)
4th quintile	22.4	23729	34	1.21 (1.16, 1.25)
5th quintile	22.3	19479	35	1.25 (1.21, 1.30)
**Region**				
London	22.4	16191	31	1.00 - Reference
South West	22.4	13488	32	1.08 (1.03, 1.14)
South Central	22.4	16958	31	1.01 (0.97, 1.05)
South East	22.4	15602	32	1.06 (1.02, 1.10)
East	22.4	14617	31	1.00 (0.97, 1.04)
West Midlands	22.5	16405	32	1.05 (1.01, 1.10)
C/N East†	22.5	21509	33	1.06 (1.01, 1.11)
North West	22.4	13354	34	1.16 (1.11, 1.22)

Overweight to obesity
N: 398 226

	BMI at baseline¶	N of transition‡	Absolute Risk(%)	Odds Ratio
**Age (in years)**				
18 - 24	27.1	8270	42	4.43 (3.89, 5.05)
25 - 34	27.2	14642	35	2.88 (2.71, 3.06)
35 - 44	27.2	24880	32	2.46 (2.35, 2.58)
45 - 54	27.3	25807	28	1.91 (1.85, 1.97)
55 - 64	27.3	21881	23	1.47 (1.43, 1.51)
65 - 74	27.3	12381	18	1.00 - Reference
**Sex**				
women	27.2	53884	28	1.00 - Reference
men	27.3	53977	27	0.96 (0.94, 0.98)
**Ethnicity**				
White	27.3	101238	27	1.00 - Reference
Mixed/Other	27.2	3246	24	0.81 (0.76, 0.86)
Asian	27.2	1274	28	0.91 (0.81, 1.02)
Black	27.3	2103	31	1.04 (0.96, 1.12)
**IMD (from less to more deprived)**				
1st quintile	27.2	23524	24	1.00 - Reference
2nd quintile	27.2	23503	26	1.06 (1.03, 1.09)
3rd quintile	27.3	22312	27	1.11 (1.07, 1.14)
4th quintile	27.3	21189	29	1.18 (1.15, 1.22)
5th quintile	27.3	17333	31	1.23 (1.19, 1.27)
**Region**				
London	27.2	12886	27	1.00 - Reference
South West	27.3	11992	27	1.07 (1.03, 1.11)
South Central	27.2	13978	26	1.02 (0.97, 1.06)
South East	27.3	13325	27	1.08 (1.04, 1.12)
East	27.2	11637	26	0.99 (0.96, 1.03)
West Midlands	27.3	13864	27	1.04 (1.00, 1.09)
C/N East†	27.3	18365	27	1.03 (0.99, 1.08)
North West	27.3	11814	29	1.16 (1.11, 1.22)

Non severe obesity to severe obesity
N: 263 736

	BMI at baseline¶	N of transition‡	Absolute Risk(%)	Odds Ratio
**Age (in years)**				
18 - 24	33.6	2707	22	5.54 (4.93, 6.23)
25 - 34	33.6	4249	15	3.33 (3.05, 3.64)
35 - 44	33.5	7031	13	2.74 (2.56, 2.95)
45 - 54	33.4	6368	10	2.05 (1.92, 2.18)
55 - 64	33.2	4611	7	1.60 (1.50, 1.72)
65 - 74	33.1	1864	5	1.00 - Reference
**Sex**				
women	33.6	16623	11	1.00 - Reference
men	33	10207	9	0.98 (0.94, 1.01)
**Ethnicity**				
White	33.4	25519	10	1.00 - Reference
Mixed/Other	33	512	8	0.77 (0.69, 0.86)
Asian	33.2	234	9	0.83 (0.67, 1.01)
Black	33.5	565	10	0.90 (0.78, 1.04)
**IMD (from less to more deprived)**				
1st quintile	33.1	4345	8	1.00 - Reference
2nd quintile	33.2	5124	9	1.09 (1.04, 1.16)
3rd quintile	33.3	5486	10	1.14 (1.08, 1.20)
4th quintile	33.5	5991	11	1.21 (1.14, 1.28)
5th quintile	33.6	5884	12	1.25 (1.18, 1.33)
**Region**				
London	33.3	3036	10	1.00 - Reference
South West	33.4	3276	10	1.05 (0.98, 1.13)
South Central	33.3	3485	10	1.02 (0.95, 1.09)
South East	33.4	3412	10	1.07 (0.98, 1.15)
East	33.3	2711	9	0.97 (0.90, 1.05)
West Midlands	33.3	3274	10	1.05 (0.98, 1.12)
C/N East†	33.4	4648	10	1.04 (0.97, 1.12)
North West	33.4	2988	11	1.15 (1.07, 1.24)

(Odds ratio axis: .5 1 2 4)

*Mutually adjusted for BMI (at baseline), age group, sex, Index of multiple deprivation (IMD – quintiles; in categories) and region
†Central/North East
‡N of individuals who transitioned to higher BMI categories
¶Mean BMI levels at baseline

Figure S15: Absolute risks and odds ratios* of transitioning at ten years from normal weight to overweight or obesity, from overweight to obesity and from non-severe to severe obesity, by age, sex, ethnicity, social deprivation and region, after excluding individuals on diuretics or with chronic diseases** at baseline

Normal weight to overweight or obesity

	BMI at baseline¶	N of transition‡	Absolute Risk(%)	Odds Ratio
**Age (in years)**				
18 - 24	21.7	14724	37	3.99 (3.57, 4.45)
25 - 34	22.1	13469	32	2.33 (2.14, 2.55)
35 - 44	22.4	15039	32	2.09 (1.92, 2.26)
45 - 54	22.6	9922	30	1.70 (1.57, 1.84)
55 - 64	22.6	4541	27	1.37 (1.25, 1.50)
65 - 74	22.6	1273	22	1.00 - Reference
**Sex**				
women	22.1	36506	30	1.00 - Reference
men	22.6	22462	37	1.14 (1.08, 1.20)
**Ethnicity**				
White	22.3	54248	32	1.00 - Reference
Mixed/Other	22.1	2717	31	0.95 (0.86, 1.04)
Asian	22.1	909	31	0.93 (0.82, 1.05)
Black	22.3	1094	37	1.15 (1.00, 1.33)
**IMD (from less to more deprived)**				
1st quintile	22.2	14322	29	1.00 - Reference
2nd quintile	22.3	13080	31	1.06 (1.00, 1.12)
3rd quintile	22.2	11791	32	1.09 (1.03, 1.17)
4th quintile	22.2	10999	35	1.21 (1.15, 1.27)
5th quintile	22.2	8776	35	1.24 (1.17, 1.31)
**Region**				
London	22.2	8015	31	1.00 - Reference
South West	22.2	5790	33	1.06 (1.00, 1.14)
South Central	22.3	7997	31	0.99 (0.93, 1.05)
South East	22.2	7398	32	1.05 (0.99, 1.11)
East	22.2	6910	30	0.98 (0.93, 1.03)
West Midlands	22.3	7091	33	1.02 (0.96, 1.08)
C/N East†	22.3	9577	34	1.05 (0.98, 1.12)
North West	22.3	6190	34	1.14 (1.06, 1.23)

Overweight to obesity

	BMI at baseline¶	N of transition‡	Absolute Risk(%)	Odds Ratio
**Age (in years)**				
18 - 24	27	5852	42	4.47 (3.88, 5.16)
25 - 34	27.1	7792	33	2.72 (2.47, 3.00)
35 - 44	27.1	10713	30	2.38 (2.15, 2.63)
45 - 54	27.1	7335	25	1.83 (1.63, 2.04)
55 - 64	27.1	3086	20	1.35 (1.21, 1.51)
65 - 74	27	796	16	1.00 - Reference
**Sex**				
women	27	18031	29	1.00 - Reference
men	27.1	17543	28	0.99 (0.95, 1.02)
**Ethnicity**				
White	27.1	32628	29	1.00 - Reference
Mixed/Other	27.1	1468	28	0.88 (0.80, 0.98)
Asian	27.1	568	30	0.90 (0.74, 1.09)
Black	27.2	910	35	1.11 (0.94, 1.31)
**IMD (from less to more deprived)**				
1st quintile	27	7879	25	1.00 - Reference
2nd quintile	27.1	7668	27	1.06 (1.02, 1.11)
3rd quintile	27.1	7216	29	1.10 (1.04, 1.16)
4th quintile	27.1	7080	31	1.18 (1.12, 1.24)
5th quintile	27.2	5731	33	1.23 (1.16, 1.31)
**Region**				
London	27.1	4662	29	1.00 - Reference
South West	27.1	3602	29	1.04 (0.97, 1.11)
South Central	27.1	4783	27	0.98 (0.91, 1.07)
South East	27.1	4459	29	1.05 (0.98, 1.12)
East	27.1	3975	27	0.97 (0.91, 1.04)
West Midlands	27.1	4125	28	1.01 (0.94, 1.10)
C/N East†	27.1	5984	30	1.03 (0.95, 1.10)
North West	27.1	3984	31	1.12 (1.04, 1.21)

Non severe obesity to severe obesity

	BMI at baseline¶	N of transition‡	Absolute Risk(%)	Odds Ratio
**Age (in years)**				
18 - 24	33.5	1680	22	6.20 (4.18, 9.18)
25 - 34	33.3	1550	13	3.39 (2.27, 5.06)
35 - 44	33.1	1857	11	2.83 (1.96, 4.09)
45 - 54	32.9	1010	8	2.09 (1.41, 3.10)
55 - 64	32.6	263	5	1.49 (0.96, 2.31)
65 - 74	32.5	41	3	1.00 - Reference
**Sex**				
women	33.3	3980	13	1.00 - Reference
men	32.8	2421	10	0.97 (0.89, 1.05)
**Ethnicity**				
White	33.1	5941	11	1.00 - Reference
Mixed/Other	32.9	193	9	0.84 (0.68, 1.02)
Asian	32.9	85	10	0.81 (0.53, 1.23)
Black	33.3	182	11	1.02 (0.77, 1.36)
**IMD (from less to more deprived)**				
1st quintile	32.9	975	9	1.00 - Reference
2nd quintile	33	1200	10	1.09 (0.93, 1.27)
3rd quintile	33.1	1331	11	1.15 (1.00, 1.32)
4th quintile	33.2	1449	12	1.24 (1.10, 1.40)
5th quintile	33.3	1446	14	1.27 (1.08, 1.49)
**Region**				
London	33.1	832	11	1.00 - Reference
South West	33.1	676	11	1.05 (0.87, 1.28)
South Central	33.1	865	11	1.05 (0.92, 1.20)
South East	33.1	820	11	1.09 (0.92, 1.29)
East	33.1	695	11	1.01 (0.86, 1.18)
West Midlands	33.1	715	11	1.06 (0.88, 1.27)
C/N East†	33.1	1085	12	1.06 (0.90, 1.24)
North West	33.1	713	12	1.16 (0.98, 1.39)

*Mutually adjusted for BMI (at baseline), age group, sex, Index of multiple deprivation (IMD – quintiles; in categories)

**Excluded: use of diuretics, prevalence of CVD, cancer, diabetes, hypertension, mental health disorders (depression, anxiety, stress, phobia, schizophrenia, bipolar disorder, affective disorder) and other chronic diseases [HIV, chronic obstructive pulmonary disease, neurological (dementia), rheumatological (rheumatoid arthritis, gout, systemic lupus erythematosus), gastro-intestinal (inflammatory bowel disease) and renal (chronic kidney disease, renal failure)]

†Central/North East

‡N of individuals who transitioned to higher BMI categories

¶Mean BMI levels at baseline

Figure S16: Absolute risks and odds ratios* of transitioning at ten years from normal weight to overweight or obesity, from overweight to obesity and from non-severe to severe obesity, by age, sex, ethnicity, social deprivation and region, after excluding individuals on diuretics or with chronic diseases** at baseline or during follow-up

Normal weight to overweight or obesity

	BMI at baseline¶	N of transition‡	Absolute Risk(%)	Odds Ratio
**Age (in years)**				
18 - 24	21.7	9377	35	3.96 (3.23, 4.85)
25 - 34	22.1	8280	30	2.23 (1.78, 2.79)
35 - 44	22.4	8468	30	2.01 (1.62, 2.48)
45 - 54	22.5	4706	27	1.61 (1.33, 1.95)
55 - 64	22.6	1590	23	1.26 (1.00, 1.59)
65 - 74	22.6	290	19	1.00 - Reference
**Sex**				
women	22	19334	27	1.00 - Reference
men	22.5	13377	36	1.18 (1.11, 1.25)
**Ethnicity**				
White	22.2	29754	30	1.00 - Reference
Mixed/Other	22	1698	30	1.00 (0.90, 1.11)
Asian	22	585	30	0.96 (0.82, 1.12)
Black	22.2	674	36	1.18 (1.01, 1.38)
**IMD (from less to more deprived)**				
1st quintile	22.2	8154	27	1.00 - Reference
2nd quintile	22.2	7338	29	1.05 (0.99, 1.12)
3rd quintile	22.2	6510	30	1.09 (1.00, 1.19)
4th quintile	22.2	6065	33	1.19 (1.10, 1.28)
5th quintile	22.1	4644	33	1.20 (1.12, 1.29)
**Region**				
London	22.1	4668	29	1.00 - Reference
South West	22.2	3125	30	1.06 (0.97, 1.16)
South Central	22.2	4559	29	0.99 (0.92, 1.06)
South East	22.1	4135	30	1.05 (0.96, 1.14)
East	22.1	3887	28	0.96 (0.90, 1.03)
West Midlands	22.2	3643	30	1.00 (0.91, 1.09)
C/N East†	22.2	5290	32	1.06 (0.97, 1.16)
North West	22.2	3404	32	1.14 (1.04, 1.24)

.5 1 2 4

Overweight to obesity

	BMI at baseline¶	N of transition‡	Absolute Risk(%)	Odds Ratio
**Age (in years)**				
18 - 24	27	3386	39	4.52 (3.48, 5.88)
25 - 34	27	4344	30	2.65 (2.06, 3.41)
35 - 44	27.1	5108	26	2.25 (1.74, 2.91)
45 - 54	27	2874	21	1.73 (1.31, 2.27)
55 - 64	27	856	17	1.27 (0.94, 1.71)
65 - 74	26.9	139	14	1.00 - Reference
**Sex**				
women	27	7910	26	1.00 - Reference
men	27.1	8797	27	1.08 (1.01, 1.14)
**Ethnicity**				
White	27	15100	26	1.00 - Reference
Mixed/Other	27	791	27	0.93 (0.82, 1.06)
Asian	27	314	29	0.98 (0.75, 1.27)
Black	27.1	502	34	1.20 (0.98, 1.46)
**IMD (from less to more deprived)**				
1st quintile	27	3698	24	1.00 - Reference
2nd quintile	27	3577	25	1.05 (0.98, 1.12)
3rd quintile	27	3405	27	1.09 (1.01, 1.17)
4th quintile	27.1	3370	29	1.16 (1.08, 1.25)
5th quintile	27.1	2657	31	1.19 (1.09, 1.30)
**Region**				
London	27	2329	27	1.00 - Reference
South West	27	1647	27	1.04 (0.93, 1.16)
South Central	27	2305	26	0.98 (0.89, 1.08)
South East	27	2115	27	1.06 (0.95, 1.18)
East	27	1874	25	0.97 (0.89, 1.07)
West Midlands	27	1738	26	1.00 (0.87, 1.15)
C/N East†	27.1	2842	28	1.04 (0.94, 1.15)
North West	27.1	1857	29	1.10 (0.99, 1.22)

.5 1 2 4

Non severe obesity to severe obesity

	BMI at baseline¶	N of transition‡	Absolute Risk(%)	Odds Ratio
**Age (in years)**				
18 - 24	33.3	749	19	5.52 (2.03, 14.97)
25 - 34	33.1	639	10	2.80 (1.05, 7.45)
35 - 44	32.9	573	8	2.25 (0.86, 5.93)
45 - 54	32.7	270	6	1.71 (0.63, 4.64)
55 - 64	32.5	47	3	0.98 (0.35, 2.78)
65 - 74	32.3	9	4	1.00 - Reference
**Sex**				
women	33.2	1311	10	1.00 - Reference
men	32.7	976	9	1.09 (0.95, 1.24)
**Ethnicity**				
White	33	2082	10	1.00 - Reference
Mixed/Other	32.8	88	9	0.94 (0.68, 1.31)
Asian	32.8	37	9	0.88 (0.40, 1.91)
Black	33.2	80	10	1.19 (0.81, 1.76)
**IMD (from less to more deprived)**				
1st quintile	32.8	342	7	1.00 - Reference
2nd quintile	32.8	411	8	1.06 (0.86, 1.30)
3rd quintile	33	465	10	1.14 (0.92, 1.41)
4th quintile	33.1	536	11	1.21 (1.01, 1.46)
5th quintile	33.2	533	13	1.25 (0.98, 1.59)
**Region**				
London	33	339	10	1.00 - Reference
South West	33	249	10	1.06 (0.80, 1.39)
South Central	33	308	9	1.05 (0.82, 1.35)
South East	33	269	9	0.99 (0.75, 1.31)
East	32.9	244	9	0.95 (0.70, 1.27)
West Midlands	32.9	235	9	1.08 (0.78, 1.48)
C/N East†	32.9	403	10	1.09 (0.82, 1.45)
North West	32.9	240	10	1.13 (0.83, 1.54)

.5 1 2 4

*Mutually adjusted for BMI (at baseline), age group, sex, Index of multiple deprivation (IMD – quintiles; in categories) and region

**Excluded: use of diuretics, prevalence of CVD, cancer, diabetes, hypertension, mental health disorders (depression, anxiety, stress, phobia, schizophrenia, bipolar disorder, affective disorder) and other chronic diseases [HIV, chronic obstructive pulmonary disease, neurological (dementia), rheumatological (rheumatoid arthritis, gout, systemic lupus erythematosus), gastro-intestinal (inflammatory bowel disease) and renal (chronic kidney disease, renal failure)]

†Central/North East

‡N of individuals who transitioned to higher BMI categories

¶Mean BMI levels at baseline

Figure S17: Absolute risks and odds ratios* of transitioning at ten years from normal weight to overweight or obesity, from overweight to obesity and from non-severe to severe obesity, by age, sex, ethnicity, social deprivation and region. BMI change was calculated without delta adjustment

Normal weight to overweight or obesity

N: 400 367

	BMI at baseline¶	N of transition‡	Absolute Risk(%)	Odds Ratio
**Age (in years)**				
18 - 24	21.7	22588	43	5.57 (5.09, 6.09)
25 - 34	22.1	26532	40	3.32 (3.14, 3.51)
35 - 44	22.4	31747	37	2.50 (2.40, 2.61)
45 - 54	22.6	26076	33	1.84 (1.76, 1.93)
55 - 64	22.8	20296	30	1.45 (1.39, 1.52)
65 - 74	22.8	11644	24	1.00 - Reference
**Sex**				
women	22.3	88559	33	1.00 - Reference
men	22.8	50324	37	1.01 (0.97, 1.05)
**Ethnicity**				
White	22.4	129685	35	1.00 - Reference
Mixed/Other	22.3	5132	34	0.93 (0.87, 0.98)
Asian	22.3	1885	36	0.94 (0.86, 1.04)
Black	22.5	2181	41	1.15 (1.05, 1.26)
**IMD (from less to more deprived)**				
1st quintile	22.4	33268	32	1.00 - Reference
2nd quintile	22.5	30837	34	1.06 (1.03, 1.09)
3rd quintile	22.4	27769	35	1.09 (1.05, 1.13)
4th quintile	22.4	25767	37	1.19 (1.15, 1.22)
5th quintile	22.3	21242	38	1.22 (1.18, 1.26)
**Region**				
London	22.4	17746	34	1.00 - Reference
South West	22.4	14515	35	1.07 (1.03, 1.11)
South Central	22.4	18363	34	1.00 (0.96, 1.03)
South East	22.4	16867	34	1.05 (1.01, 1.09)
East	22.4	15907	34	0.99 (0.96, 1.03)
West Midlands	22.5	17650	35	1.03 (0.99, 1.07)
C/N East†	22.5	23303	36	1.04 (0.99, 1.09)
North West	22.4	14532	37	1.16 (1.10, 1.21)

.5 1 2 4

Overweight to obesity

N: 398 226

	BMI at baseline¶	N of transition‡	Absolute Risk(%)	Odds Ratio
**Age (in years)**				
	27.1	9635	49	6.09 (5.40, 6.87)
	27.2	17267	41	3.87 (3.63, 4.14)
	27.2	26671	34	2.78 (2.66, 2.90)
	27.3	26111	28	1.95 (1.87, 2.04)
	27.3	22003	23	1.48 (1.44, 1.53)
	27.3	12445	18	1.00 - Reference
**Sex**				
	27.2	59306	30	1.00 - Reference
	27.3	54826	27	0.88 (0.86, 0.90)
**Ethnicity**				
	27.3	106893	29	1.00 - Reference
	27.2	3531	27	0.84 (0.78, 0.89)
	27.2	1396	31	0.93 (0.82, 1.04)
	27.3	2312	35	1.07 (0.97, 1.17)
**IMD (from less to more deprived)**				
	27.2	24757	26	1.00 - Reference
	27.2	24779	27	1.06 (1.02, 1.09)
	27.3	23543	29	1.10 (1.07, 1.13)
	27.3	22495	31	1.17 (1.14, 1.21)
	27.3	18558	33	1.21 (1.17, 1.25)
**Region**				
	27.2	13746	28	1.00 - Reference
	27.3	12639	29	1.06 (1.02, 1.11)
	27.2	14780	28	1.01 (0.96, 1.05)
	27.3	14034	29	1.07 (1.03, 1.11)
	27.2	12313	28	0.99 (0.95, 1.02)
	27.3	14658	28	1.03 (0.98, 1.07)
	27.3	19466	29	1.02 (0.97, 1.06)
	27.3	12496	31	1.15 (1.11, 1.20)

.5 1 2 4

Non severe obesity to severe obesity

N: 263 736

	BMI at baseline¶	N of transition‡	Absolute Risk(%)	Odds Ratio
**Age (in years)**				
	33.6	3243	26	7.90 (7.05, 8.85)
	33.6	5228	19	4.55 (4.13, 5.03)
	33.5	7668	14	3.14 (2.91, 3.38)
	33.4	6544	10	2.14 (2.00, 2.29)
	33.2	4640	8	1.62 (1.51, 1.73)
	33.1	1916	5	1.00 - Reference
**Sex**				
	33.6	18698	13	1.00 - Reference
	33	10541	9	0.90 (0.87, 0.93)
**Ethnicity**				
	33.4	27753	11	1.00 - Reference
	33	572	9	0.81 (0.71, 0.92)
	33.2	271	10	0.86 (0.71, 1.03)
	33.5	643	12	0.94 (0.83, 1.08)
**IMD (from less to more deprived)**				
	33.1	4699	9	1.00 - Reference
	33.2	5561	10	1.09 (1.03, 1.15)
	33.3	5974	11	1.12 (1.07, 1.18)
	33.5	6541	12	1.19 (1.13, 1.26)
	33.6	6464	14	1.24 (1.17, 1.31)
**Region**				
	33.3	3308	11	1.00 - Reference
	33.4	3574	11	1.05 (0.98, 1.12)
	33.3	3803	11	1.01 (0.94, 1.09)
	33.4	3699	11	1.06 (0.98, 1.15)
	33.3	2973	10	0.96 (0.90, 1.04)
	33.3	3558	11	1.03 (0.96, 1.12)
	33.4	5049	11	1.03 (0.96, 1.10)
	33.4	3275	12	1.15 (1.07, 1.23)

.5 1 2 4

* Mutually adjusted for BMI (at baseline), age group, sex, Index of multiple deprivation (IMD – quintiles; in categories), ethnicity, region, use of diuretics, prevalence of CVD, cancer, diabetes, hypertension, mental health disorders (depression, anxiety, stress, phobia, schizophrenia, bipolar disorder, affective disorder) and other chronic diseases [HIV, chronic obstructive pulmonary disease, neurological (dementia), rheumatological (rheumatoid arthritis, gout, systemic lupus erythematosus), gastro-intestinal (inflammatory bowel disease) and renal (chronic kidney disease, renal failure)]

†Central/North East

‡N of individuals who transitioned to higher BMI categories

¶Mean BMI levels at baseline

Figure S18: Absolute risks and odds ratios* of transitioning at ten years from normal weight to overweight or obesity, from overweight to obesity and from non-severe to severe obesity, by age, sex, ethnicity, social deprivation and region. Results from the complete case analysis

Normal weight to overweight or obesity

	BMI at baseline¶	N of transition‡	Absolute Risk(%)	Odds Ratio
**Age (in years)**				
18 - 24	21.7	3998	38	4.14 (3.87, 4.44)
25 - 34	22.1	6818	37	3.04 (2.87, 3.22)
35 - 44	22.4	10875	36	2.58 (2.45, 2.72)
45 - 54	22.6	10407	34	2.02 (1.92, 2.12)
55 - 64	22.8	9624	31	1.51 (1.44, 1.58)
65 - 74	22.8	6118	24	1.00 - Reference
**Sex**				
women	22.3	32403	32	1.00 - Reference
men	22.9	15437	35	1.05 (1.02, 1.09)
**Ethnicity**				
White	22.5	38218	33	1.00 - Reference
Mixed/Other	22.5	1215	32	0.85 (0.78, 0.93)
Asian	22.4	352	32	0.94 (0.81, 1.09)
Black	22.6	490	42	1.22 (1.06, 1.41)
**IMD (from less to more deprived)**				
1st quintile	22.6	8974	29	1.00 - Reference
2nd quintile	22.6	8521	32	1.11 (1.07, 1.16)
3rd quintile	22.5	7740	33	1.18 (1.13, 1.23)
4th quintile	22.5	7368	36	1.37 (1.31, 1.43)
5th quintile	22.4	6291	37	1.47 (1.40, 1.54)
**Region**				
London	22.5	5284	32	1.00 - Reference
South West	22.5	4986	34	1.08 (1.01, 1.14)
South Central	22.5	6213	31	1.01 (0.95, 1.07)
South East	22.5	5702	32	1.07 (1.01, 1.13)
East	22.5	5377	31	1.02 (0.96, 1.08)
West Midlands	22.6	6751	33	1.05 (1.00, 1.12)
C/N East†	22.6	8592	34	1.05 (0.99, 1.10)
North West	22.5	4935	37	1.22 (1.15, 1.30)

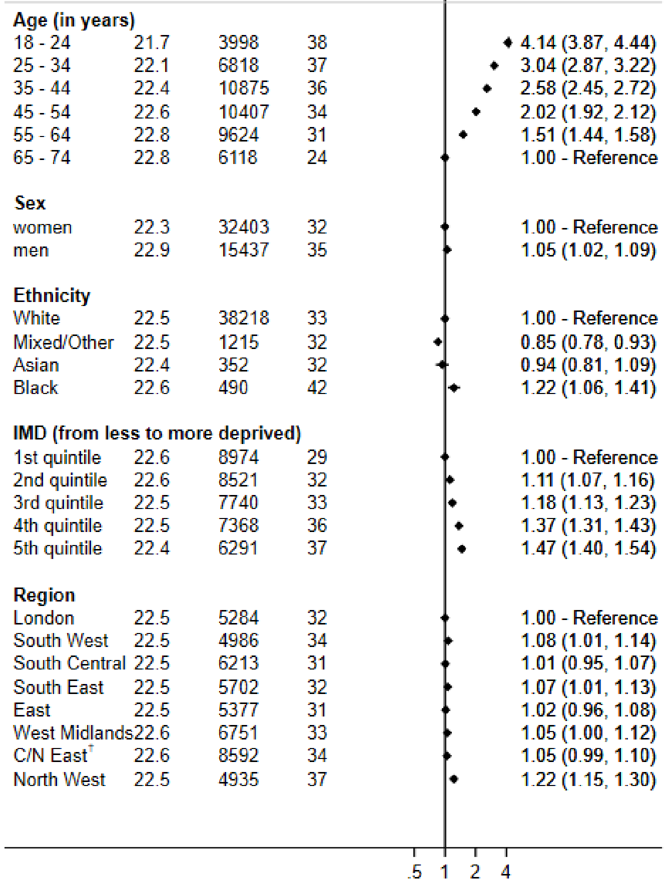

Overweight to obesity

	BMI at baseline¶	N of transition‡	Absolute Risk(%)	Odds Ratio
**Age (in years)**				
18 - 24	27.1	1913	46	1.48 (1.38, 1.58)
25 - 34	27.2	4879	41	1.73 (1.65, 1.82)
35 - 44	27.3	9258	34	1.68 (1.61, 1.76)
45 - 54	27.3	10700	28	1.54 (1.48, 1.60)
55 - 64	27.3	10885	23	1.41 (1.36, 1.47)
65 - 74	27.3	6680	17	1.00 - Reference
**Sex**				
women	27.2	25226	30	1.00 - Reference
men	27.4	19089	23	0.73 (0.71, 0.75)
**Ethnicity**				
White	27.3	36369	27	1.00 - Reference
Mixed/Other	27.2	922	22	0.69 (0.64, 0.74)
Asian	27.2	317	29	0.75 (0.66, 0.85)
Black	27.4	535	31	0.74 (0.67, 0.83)
**IMD (from less to more deprived)**				
1st quintile	27.2	7671	23	1.00 - Reference
2nd quintile	27.3	7786	25	1.04 (1.00, 1.08)
3rd quintile	27.3	7767	27	1.15 (1.11, 1.19)
4th quintile	27.3	7361	29	1.23 (1.18, 1.27)
5th quintile	27.4	6476	32	1.33 (1.28, 1.38)
**Region**				
London	27.3	4698	26	1.00 - Reference
South West	27.3	4899	27	1.07 (1.02, 1.13)
South Central	27.3	5731	26	1.09 (1.04, 1.15)
South East	27.3	5229	27	1.10 (1.05, 1.16)
East	27.3	4604	25	1.04 (0.99, 1.10)
West Midlands	27.3	6347	26	1.24 (1.19, 1.30)
C/N East†	27.3	8170	27	1.15 (1.09, 1.20)
North West	27.3	4637	29	1.09 (1.03, 1.14)

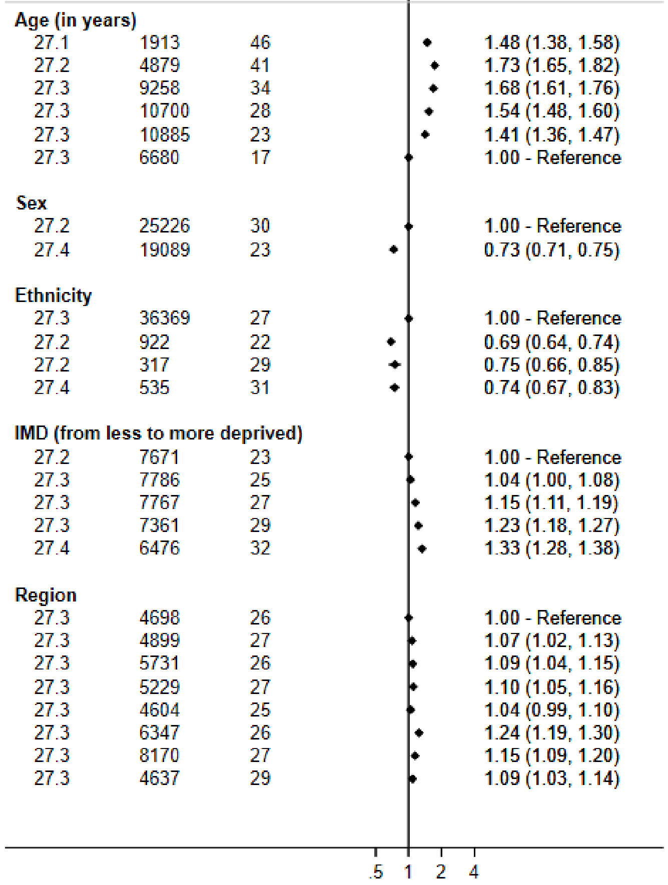

Non severe obesity to severe obesity

	BMI at baseline¶	N of transition‡	Absolute Risk(%)	Odds Ratio
**Age (in years)**				
18 - 24	33.7	776	26	2.52 (2.24, 2.83)
25 - 34	33.8	1852	20	2.63 (2.39, 2.88)
35 - 44	33.7	3383	15	2.49 (2.29, 2.70)
45 - 54	33.5	3431	11	2.04 (1.88, 2.20)
55 - 64	33.3	2755	8	1.71 (1.59, 1.85)
65 - 74	33.1	1169	5	1.00 - Reference
**Sex**				
women	33.7	8889	13	1.00 - Reference
men	33.1	4477	8	0.81 (0.77, 0.84)
**Ethnicity**				
White	33.5	11341	11	1.00 - Reference
Mixed/Other	33.1	169	7	0.59 (0.50, 0.70)
Asian	33.2	64	8	0.60 (0.46, 0.78)
Black	33.6	175	11	0.61 (0.52, 0.73)
**IMD (from less to more deprived)**				
1st quintile	33.2	1739	9	1.00 - Reference
2nd quintile	33.3	2135	10	1.10 (1.03, 1.18)
3rd quintile	33.4	2379	11	1.20 (1.12, 1.29)
4th quintile	33.6	2626	12	1.28 (1.20, 1.37)
5th quintile	33.7	2603	14	1.31 (1.22, 1.40)
**Region**				
London	33.4	1421	11	1.00 - Reference
South West	33.5	1556	11	0.98 (0.90, 1.07)
South Central	33.4	1751	11	1.11 (1.02, 1.21)
South East	33.5	1660	11	1.11 (1.02, 1.21)
East	33.4	1320	10	0.99 (0.91, 1.09)
West Midlands	33.3	1771	10	1.19 (1.09, 1.29)
C/N East†	33.4	2460	11	1.13 (1.04, 1.22)
North West	33.5	1427	12	1.07 (0.98, 1.17)

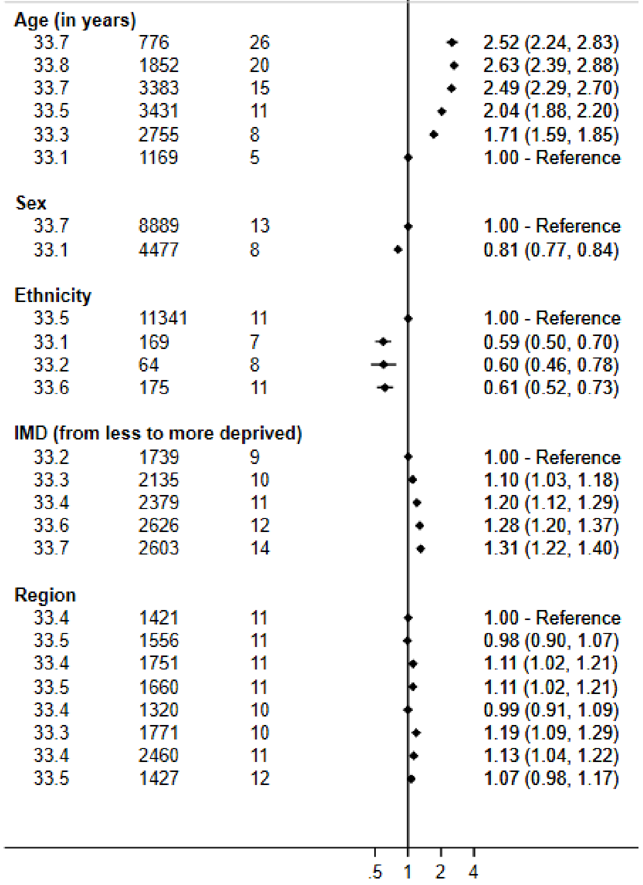

* Mutually adjusted for BMI (at baseline), age group, sex, Index of multiple deprivation (IMD – quintiles; in categories), ethnicity, region, use of diuretics, prevalence of CVD, cancer, diabetes, hypertension, mental health disorders (depression, anxiety, stress, phobia, schizophrenia, bipolar disorder, affective disorder) and other chronic diseases [HIV, chronic obstructive pulmonary disease, neurological (dementia), rheumatological (rheumatoid arthritis, gout, systemic lupus erythematosus), gastro-intestinal (inflammatory bowel disease) and renal (chronic kidney disease, renal failure)]

†Central/North East

‡N of individuals who transitioned to higher BMI categories

¶Mean BMI levels at baseline

Table S16: Odds ratios* of transitioning at ten years from i) normal weight (BMI≥18.5 & BMI<25kg/m^2) to overweight or obesity (BMI≥25kg/m^2), ii) overweight (BMI≥25 & BMI<30kg/m^2) to obesity and iii) non-severe (BMI≥30 & BMI<40kg/m^2) to severe obesity (BMI≥40kg/m^2), by age, sex, ethnicity, social deprivation, region, separately in never, former and current smokers

	**From normal weight to overweight/ obesity**			**From overweight to obesity**			**From non-severe to severe obesity**		
	Never smoker	Former smoker	Current smoker	Never smoker	Former smoker	Current smoker	Never smoker	Former smoker	Current smoker
**Age**									
18-24yo	4.71 (4.28-5.18)	3.95 (3.56-4.39)	4.85 (4.24-5.56)	5.25 (4.60-5.99)	4.39 (3.81-5.05)	5.46 (4.59-6.49)	6.69 (5.57-8.03)	6.03 (5.18-7.01)	6.47 (4.89-8.57)
25-34yo	2.66 (2.42-2.92)	2.55 (2.37-2.74)	3.00 (2.68-3.35)	3.21 (2.95-3.50)	2.94 (2.70-3.22)	3.48 (3.03-4.00)	3.70 (3.26-4.19)	3.75 (3.30-4.26)	3.81 (2.89-5.03)
35-44yo	2.32 (2.21-2.45)	2.41 (2.24-2.60)	2.51 (2.22-2.83)	2.65 (2.47-2.84)	2.65 (2.47-2.84)	2.84 (2.50-3.22)	2.99 (2.65-3.38)	2.98 (2.67-3.33)	3.18 (2.46-4.10)
45-54yo	1.82 (1.72-1.92)	1.99 (1.86-2.12)	1.97 (1.75-2.21)	1.96 (1.84-2.09)	2.07 (1.95-2.20)	2.11 (1.85-2.39)	2.13 (1.92-2.36)	2.28 (2.06-2.54)	2.15 (1.67-2.78)
55-64yo	1.41 (1.33-1.49)	1.57 (1.48-1.68)	1.59 (1.39-1.74)	1.43 (1.34-1.52)	1.53 (1.46-1.61)	1.64 (1.48-1.83)	1.50 (1.35-1.67)	1.73 (1.58-1.90)	1.86 (1.45-2.38)
65-74yo	ref	ref	ref	ref	ref	ref	ref	ref	ref
**Sex**									
Men	1.12 (1.07-1.18)	1.13 (1.08-1.19)	1.09 (1.05-1.14))	0.95 (0.92-0.98)	0.96 (0.93-0.99)	1.04 (0.99-1.09)	1.00 (0.95-1.06)	0.96 (0.91-1.01)	1.04 (0.94-1.15)
Women	ref	ref	ref	ref	ref	ref	ref	ref	ref
**IMD**									
1st quintile	ref	ref	ref	ref	ref	ref	ref	ref	ref
2nd quintile	1.06 (1.02-1.10)	1.09 (1.04-1.14)	1.02 (0.95-1.10)	1.06 (1.01-1.12)	1.05 (1.01-1.09)	1.06 (0.97-1.15)	1.10 (1.02-1.19)	1.09 (1.00-1.19)	1.10 (0.90-1.34)
3rd quintile	1.10 (1.04-1.17)	1.11 (1.06-1.16)	1.05 (0.97-1.14)	1.10 (1.06-115)	1.12 (1.07-1.17)	1.10 (1.02-1.18)	1.13 (1.04-1.22)	1.19 (1.06-1.32)	1.03 (0.86-1.25)
4th quintile	1.21 (1.14-1.27)	1.24 (1.18-1.30)	1.15 (1.06-1.24)	1.17 (1.11-1.24)	1.21 (1.15-1.27)	1.18 (1.09-1.27)	1.17 (1.08-1.26)	1.27 (1.15-1.41)	1.17 (0.97-1.42)
5th quintile	1.26 (1.21-1.32)	132 (1.24-1.40)	1.19 (1.09-1.29)	1.24 (1.18-1.31)	1.29 (1.23-1.35)	1.19 (1.09-1.30)	1.23 (1.12-1.34)	1.34 (1.21-1.48)	1.19 (1.03-1.39)
**Ethnicity**									
White	ref	ref	ref	ref	ref	ref	ref	ref	ref
Mixed/ Other	0.94 (0.87-1.02)	0.85 (0.72-0.99)	0.97 (0.81-1.15)	0.84 (0.78-0.90)	0.79 (0.68-0.92)	0.91 (0.74-1.11)	0.83 (0.72-0.95)	0.64 (0.48-0.86)	0.85 (0.59-1.21)
Asian	1.14 (1.00-1.29)	1.15 (0.98-1.34)	1.12 (0.93-1.35)	1.06 (0.97-1.15)	1.04 (0.88-1.21)	1.05 (0.80-1.37)	0.97 (0.80-1.17)	0.82 (0.65-1.03)	0.88 (0.64-1.23)
Black	0.95 (0.85-1.07)	0.90 (0.75-1.08)	0.91 (0.76-1.09)	0.95 (0.81-1.11)	0.91 (0.79-1.05)	0.86 (0.66-1.12)	0.84 (0.61-1.17)	0.77 (0.56-1.06)	0.92 (0.58-1.46)

*Mutually adjusted for BMI (at baseline), age group, sex, Index of multiple deprivation (IMD – quintiles; in categories), ethnicity, region, use of diuretics, prevalence of CVD, cancer, diabetes, hypertension, mental health disorders (dep depression, anxiety, stress, phobia, schizophrenia, bipolar disorder, affective disorder) and other chronic diseases [HIV, chronic obstructive pulmonary disease, neurological (dementia), rheumatological (rheumatoid arthritis, gout, systemic lupus erythematosus), gastro-intestinal (inflammatory bowel disease) and renal (chronic kidney disease, renal failure)]

Table S17: Odds ratios* of transitioning at ten years from i) normal weight (BMI≥18.5 & BMI<25kg/m^2) to overweight or obesity (BMI≥25kg/m^2), ii) overweight (BMI≥25 & BMI<30kg/m^2) to obesity and iii) non-severe (BMI≥30 & BMI<40kg/m^2) to severe obesity (BMI≥40kg/m^2), by age, sex, ethnicity, social deprivation, region and smoking status

	**From normal weight to overweight/ obesity**	**From overweight to obesity**	**From non-severe to severe obesity**
**Age**			
18-24yo	4.52 (4.13-4.94)	5.03 (4.45-5.70)	6.45 (5.76-7.22)
25-34yo	2.68 (2.53-2.85)	3.17 (2.98-3.38)	3.74 (3.40-4.11)
35-44yo	2.37 (2.28-2.47)	2.67 (2.55-2.80)	3.03 (2.81-3.27)
45-54yo	1.89 (1.81-1.97)	2.02 (1.94-2.10)	2.19 (2.05-2.35)
55-64yo	1.49 (1.43-1.56)	1.50 (1.46-1.55)	1.65 (1.54-1.77)
65-74yo	ref	Ref	ref
**Sex**			
Men	1.13 (1.09-1.16)	0.97 (0.95-0.99)	0.99 (0.96-1.03))
Women	ref	ref	Ref
**IMD**			
1st quintile	ref	ref	Ref
2nd quintile	1.07 (1.04-1.10)	1.06 (1.03-1.09)	1.10 (1.04-1.16)
3rd quintile	1.10 (1.06-1.14)	1.11 (1.08-1.14)	1.14 (1.08-1.20)
4th quintile	1.21 (1.17-1.26)	1.19 (1.16-1.23)	1.22 (1.14-1.29)
5th quintile	1.27 (1.23-1.31)	1.25 (1.22-1.29)	1.27 (1.20-1.35)
**Ethnicity**			
White	ref	Ref	ref
Mixed/Other	0.92 (0.87-0.98)	0.84 (0.79-0.90)	0.80 (0.71-0.89)
Asian	1.14 (1.04-1.25)	1.06 (0.98-1.14)	0.92 (0.79-1.06)
Black	0.93 (0.86-1.01)	0.92 (0.82-1.04)	0.84 (0.68-1.02)
**Smoking status**			
Never	ref	ref	ref
Former	1.24 (1.21-1.27)	1.24 (1.22-1.27)	1.21 (1.16-1.27)
Current	0.85 (0.82-0.88)	0.82 (0.80-0.85)	0.81 (0.78-0.85)

*Mutually adjusted for BMI (at baseline), age group, sex, Index of multiple deprivation (IMD – quintiles; in categories), ethnicity, region, use of diuretics, prevalence of CVD, cancer, diabetes, hypertension, mental health disorders (dep depression, anxiety, stress, phobia, schizophrenia, bipolar disorder, affective disorder) and other chronic diseases [HIV, chronic obstructive pulmonary disease, neurological (dementia), rheumatological (rheumatoid arthritis, gout, systemic lupus erythematosus), gastro-intestinal (inflammatory bowel disease) and renal (chronic kidney disease, renal failure)] and smoking status
